# Proceedings of the Sixteenth International Society of Sports Nutrition (ISSN) Conference and Expo: Las Vegas, NV, USA. 13-15 June 2019

**DOI:** 10.1186/s12970-020-00352-x

**Published:** 2020-05-31

**Supplement Editor name** Trisha A. VanDusseldorp

I**nstitution/department** Kennesaw State University, Wellstar College of Health & Human Services, Dept. Exercise Science & Sport Management

**Street** 520 Parliament Garden Way NW

**City** Kennesaw

**Post code** GA 30144

**Country** USA

**Email adress** tvanduss@kennesaw.edu

**Supplement Editor name** Douglas S. Kalman

**Institution/department** Scientifc Affairs, Nutrasource Diagnostics, Inc

**Street** 120 Research Lane

**City** Guelph

**Post code** N1G 0B4

**Country** ON, Canada

**Email adress** dkalman@nutrasource.ca

**Supplement Editor name** Shawn M. Arent

**Institution/department** University of South Carolina, Exercise Science

**Street** 921 Assembly Street

**City** Columbia

**Post code** SC 29208

**Country** USA

**Email adress** sarent@mailbox.sc.edu

**Supplement Editor name** Jose Antonio

**Institution/department** Nova Southeastern University, Exercise and Sport Sciences

**Street** 3532 S. University Drive

**City** Davie

**Post code** FL 33314

**Country** USA

**Email adress** ja839@nova.edu

## A1 MMA fighters and epigenetics: An analysis of miRNA expression and global DNA methylation

### Dominick Cabrera^1^, Ashley Almanzar^3^, Julius D. Thomas^1^, Jose Antonio^2^, Corey A. Peacock^2^, Jaime L. Tartar^1^

#### ^1^Nova Southeastern University, College of Psychology, Ft. Lauderdale, FL USA; ^2^Nova Southeastern University, College of Health Care Sciences, Ft. Lauderdale, FL USA; ^3^Miami Dade College, College of Science, Miami, FL USA

##### **Correspondence:** Jaime L. Tartar (tartar@nova.edu)

**Background**

Psychological and physical stress can induce dysregulation of gene expression via changes in methylation and miRNA expression. Such epigenetic modifications are yet to be investigated in professional MMA fighters who are subject to highly stressful training involving repeated head trauma. DNA methylation changes associate with a variety of health markers (e.g. chronic inflammation, autoimmune disease, and various cancers). The miRNAs that will be assessed have been linked to various autoimmune diseases and cancers. The fighters tested in the current study train at the highest level of striking and grappling, often following hypocaloric diets in preparation for professional competition in front of a global viewing audience. The proposed study will examine differences in DNA methylation and miRNA expression in elite MMA fighters compared to active controls.

**Materials and Methods**

RNA was extracted from plasma samples of 20 fighters and 20 controls using a QIAcube instrument (QIAGEN, Valencia, CA, USA). The RNA was converted to cDNA and these samples were pooled by groups (MMA and control) for a PCR array to determine specific miRNAs with differential expression. Real time PCR quantified the expression of these miRNAs in individuals of both groups using QIAGEN kits. Global methylation differences between groups was also assessed via a LINE-1 assay (surrogate global DNA analysis), a product of Active Motif (Carlsbad, CA, USA).

**Results**

This study is currently ongoing. We show pooled and individual miRNA expression and LINE-1 methylation between the MMA and control groups.

**Conclusions**

A significant difference in miRNA expression or DNA methylation between MMA fighters and control athletes would indicate that fighters are at risk for developing certain diseases as a result of the stress associated with their training. Identifying which specific miRNAs are differentially expressed offers potential therapeutic targets for the treatment of fighters suffering from illness. A difference in methylation offers insight into the mechanism underlying the phenotypic differences these fighters experience. No significant difference in the epigenetic modifications assessed between these two groups could indicate that despite the stress of MMA training, these fighters are not at any greater risk of developing diseases associated with differential methylation or expression of the specific miRNAs tested. This contributes to the growing body of literature on epigenetic modifications associated with traumatic brain injury experienced in athletic competition.

**Support**

This study is supported by a Nova Southeastern University 2019-2020 President’s Faculty Research and Development Grant to JLT, CP, JA.

## A2 No Benefits to a Fast Fast: A 13 Hour Fast Decreases Cortisol, Increases Inflammation, and Increases Mind Wandering,

### Jaime L. Tartar^1^, Kayla Thompson^1^, Federico Earhart^2^, Jonathan B. Banks^1^

#### ^1^Nova Southeastern University, College of Psychology, Ft. Lauderdale, FL, USA; ^2^Miami Dade College, College of Science, Miami, FL, USA

##### **Correspondence:** Jaime L. Tartar (tartar@nova.edu)

**Background**

A large and growing body of research has shown that long-term fasting (>24 h) or sustained intermittent fasting can reduce inflammation and mediate improved cognitive and psychological health. The effects of short-term fasting on these processes are not well-understood. In order to begin to address this uncertainty, we investigated the possibility that a short-term (13 hour) fast could increase cognitive processes, and reduce inflammation.

**Materials and Methods**

We applied a crossover design study, with a baseline and a fasting session occurring one week apart (condition order was counterbalanced). For the fasting session, participants were instructed not to eat for at least 13 hours before the study session. For the baseline session, participants were instructed to eat breakfast. The cognitive test battery included measures of working memory, sustained attention, speed of processing, cognitive inhibition, and mind wandering. Blood and saliva samples were obtained at the end of each session for energy and stress (cortisol) and inflammation (IL-1β and Il-6) biomarker quantification. Height, weight, heart rate, and blood pressure were also recorded.

**Results**

Cognition data: Data from 4 participants were removed due to failure to complete one or more cognitive tasks as instructed. No significant difference was observed on the working memory task (Symmetry Span), speed of processing (Pattern comparison), inhibition (Flanker), or sustained attention (SART target accuracy and dprime), all *p*’s > .05. Mind wandering was higher at fasting (*M* = .60, *SD* = .28) than at baseline (*M* = .53, *SD* = .27), *t*(29) = 2.27, *p* = .031, *d* = 0.25. False alarms on the sustained attention task were higher during fasting (*M* = 16.54, *SD* = 9.73) than during baseline (*M* = 14.72, *SD* = 10.05), t (29) = 2.22, p = .035, *d* = .18.

Biomarker Data: Cortisol levels were significantly higher at fasting (*M* = .15 μg/dL *SD* = .06) than at baseline (*M* = .19 μg/dL, *SD* = .07), *t*(32) = 3.16, *p* = .003, *d* = 0.25. IL-1β levels were significantly higher at fasting (*M* = 29.57 pg/mL *SD* = 23.51) than at baseline (*M* = 21.24 pg/mL, *SD* = 17.55), *t*(32) = -2.44, *p* = .02. There was not a significant difference in IL-6 levels between fasting (*M* = 6.06 pg/mL *SD* = 7.05) and baseline (*M* = 7.89 pg/mL, *SD* = 11.56), *p >* .05.

**Conclusions**

Overall, our findings show that a short-term fast does not benefit cognitive processes, and in fact, results in an increase in mind wandering and an increase in false alarms during an attention task. In addition, our data suggest that short-term fasting can alter cortisol and inflammation due to short-term changes in energy demands and that anti-inflammatory processes and beneficial cognitive changes likely require, either a longer fasting or intermittent fasting.

**Support**

This study was supported by a Department of Education sub-contract award to JLT (P031C160143).

## A3 The Influence of When You Exercise or Exercise Intensity on Indices of Sleep Quality

### Victoria Burgess^1,2^, Sarah Knafo^1^, Jose Antonio^2^, Jaime Tartar^1^

#### ^1^Department of Psychology and Neuroscience, NSU Florida, Davie, FL USA; ^2^Exercise and Sport Science, NSU Florida, Davie, FL USA

##### **Correspondence:** Jose Antonio (ja839@nova.edu)

**Background**

Sleep is an extremely important component of health and exercise training/performance. Recent studies have highlighted the interaction between sleep, recovery, and performance in elite and recreational athletes. Thus, the purpose of this study was to examine whether the time of exercise or intensity of exercise affected sleep quality in active individuals.

**Materials and Methods**

Thirty four active individuals, men (n=11) and women (n=23) (mean±SD: age 39±11 years; height 169.2±10.4 centimeters; weight; 72.8±10.9 kilograms), were self-grouped into an AM (before 11:59am) or PM (12-11:59pm) exercise group and a exercise intensity group of either moderate (MOD) (150 min/wk) or vigorous (VIG) (75 min/wk) intensity exercise based on a specific set of criteria. They participated in a seven-day quantitative, quasi-experimental, exploratory study, wearing an Actigraph watch. Four measures of sleep were measured through the Actigraph watch: total sleep time (TST), sleep onset latency (SOL), sleep efficiency % (SE), and wake after sleep onset (WASO). Data was analyzed using a factorial ANOVA to examine the relationship between exercise timing and intensity on sleep quality of the participants.

**Results**

There were no significant differences in sleep quality or duration in either the time group (AM vs. PM) or the intensity group (MOD vs. VIG). Results within the AM and PM group results showed no significant differences (Mean±SD: TST – AM 6.7±0.9, PM 6.8±0.9. SOL – AM 14.9±9.2, PM 14.4±11.6. SE - AM 84.2±4.6, PM 84.7±4.2. WASO – AM 44.1±13.3, PM 43.2±12.4. Further, no significant differences were found within the intensity group (MOD and VIG): Mean±SD: total sleep time hours – MOD 6.8±0.8, VIG 6.6±0.9; SOL – MOD 14.9±11.8, VIG 14.5±9.1. Sleep Efficiency % - MOD 85.0±4.3, VIG 84.0±4.5. WASO – MOD 42.4±12.2, VIG 45.0±13.4

**Conclusions**

Based on this cross-sectional data, there is no relationship between exercise intensity or the time of exercise on indices of sleep quality or duration.

## A4 The Effects of HMB Supplementation on Body Composition in MMA Fighters

### Denvyr Tyler-Palmer^1^, Corey A. Peacock^1^, Jamie Tartar^2^, Tobin Silver^1^, Jose Antonio^1^

#### ^1^Exercise and Sport Science, Nova Southeastern University, Davie, FL USA; ^2^Psychology, Nova Southeastern University, Davie, FL USA

##### **Correspondence:** Jose Antonio (ja839@nova.edu)

**Background**

Prior research has shown that HMB supplementation may have a positive effect on body composition; however, minimal research exists regarding its effects in Mixed Martial Arts (MMA) Fighters. Therefore, the purpose of the current study is to determine the effects of HMB supplementation on body composition in MMA fighters.

**Materials and Methods**

Sixteen competitive, healthy MMA fighters (29±3.5 yrs.; 178.5± 7.8 cm; males) completed a double-blinded, counterbalanced, two condition [HMB versus Placebo (Cellulose)] by two-time point [Pre-, Post-] study. The study consisted of subjects supplementing HMB (3 g daily) or placebo in conjunction with MMA training over a 6-week period. Body composition was assessed via the In-Body770® both pre- and post-intervention.

**Results**

There were no significant (p =0.471) differences between HMB and Placebo following the 6-weeks of training for weight (kilograms [kg]) (HMB Pre- 84.6±10.8, Post- 84.1±11.6; Placebo Pre- 87.9±14.2, Post- 87.9±13.5). There were also no significant (p = 0.095) differences for fat-free mass (kg) (HMB Pre- 42.3±5.4, Post- 41.8±5.1; Placebo Pre- 44.2±9.0, Post- 44.6±8.8). No significant (p = 0.655) differences existed for fat mass (kg) (HMB Pre- 11.3±2.5, Post- 11.2±3.8; Placebo Pre- 11.0±4.9, Post- 11.2±3.6). And finally no differences (p = 0.641) were found for percent body fat (HMB Pre- 13.3±2.5, Post- 13.2±3.4; Placebo Pre- 12.8±6.4, Post- 12.3±6.6).

**Conclusion**

Based on this pilot study, HMB supplementation had no effect on body composition over a 6-week period in MMA fighters. A study utilizing a larger sample coupled with an aggressive strength-training program may be warranted.

## A5 Neurobehavioral Assessment in Active and Recently Retired NFL Players and MMA fighters

### Julius D. Thomas^1^, Dominick Cabrera^1^, Jose Antonio^2^, Corey A. Peacock^2^, Jaime L. Tartar^1^

#### ^1^Nova Southeastern University, College of Psychology, Ft. Lauderdale, FL USA; ^2^Nova Southeastern University, College of Health Care Sciences, Ft. Lauderdale, FL USA

##### **Correspondence:** Jaime L. Tartar (tartar@nova.edu)

**Background**

Professional contact sport athletes are at risk for developing Chronic Traumatic Encephalopathy (CTE) - a degenerative brain disease caused by repetitive head impacts. Until very recently, CTE was not well-understood and was initially observed in boxers and classified as “dementia pugilistica”. Due to the robust relationship between mood and long-term outcomes for neurological health in contact sport athletes, we tested two groups of elite contact sports athletes, NFL players and professional MMA fighters, on a comprehensive set of neurobehavioral health which included both emotion and cognition domains and compared these groups to a professional non-control sport control group.

**Materials and Methods**

This study used a cross sectional design and included only males. We tested 9 active or recently retired NFL players (M age = 31, SD = 4.3), 10 active or recently retired professional MMA fighters (M age = 29.6, SD = 1.65), and a control group that consisted of 12 professional non-contact sport athletes (e.g. runners, swimmers etc..) (M age = 39, SD = 12.5). In order to control for diurnal influences on our biomarker measures, blood and saliva collection took place between noon-6:00 p.m. Cognition Testing was carried out using The NIH Toolbox Cognition battery and included the Flanker Task (*Executive Function & Attention*), The List Sorting Task (working Memory), The Dimensional Change Card Sort Test (*Executive Function*), and The Pattern Comparison Task (*Processing Speed*). Emotion was Assessed using test The NIH Toolbox Emotion measures and included all four major domains: Psychological Well-Being, Stress and Self-Efficacy, Social Relationships and Negative Affect. Findings of the cognition and emotion measures were related to biomarkers of stress (cortisol) and inflammation (IL-6 and IL-1β).

**Results**

There were no significant differences on cognitive or emotion measure between the MMA group and the control group on any cognitive or emotion measure (all p’s > 0.05). Compared to the control group, the NFL group showed significant differences on 4 emotion measures. Perceived stress was higher in the NFL group (*M* =50, *SD* = 11.87) compared to the control group (*M* = 41, *SD* = 5.45), *t*(19) = 2.24, *p* = 0.04. Fear was higher in the NFL group (*M* = 56.11, *SD* = 10.776) compared to the control group (*M* = 47.25, *SD* = 6.14), *t*(19) = 2.39, *p* = 0.03. Anxiety/fear was higher in the NFL group (*M* = 56.11, *SD* = 10.776) compared to the control group (*M* = 47.25, *SD* = 6.14), *t*(19) = 2.39, *p* = 0.03. Relatedly, perceptions of physical anxiety (somatic arousal) was higher in the NFL group (*M* = 56.11, *SD* = 10.776) compared to the control group (*M* = 47.25, *SD* = 6.14), *t*(19) = 2.39, *p* = 0.03. Finally, perceptions of anger and physical aggression were significantly higher in the NFL group (*M* = 61.67, *SD* = 6.60), compared to the control group (*M* = 51.92, *SD* = 8.37), *t*(19) = 2.88, *p* = 0.01. There were no significant differences between the NFL group and the control group on cognitive measures (all p’s > 0.05). Follow up correlations between the significant psychological measures and biomarkers in the NFL group showed that the pro-inflammatory cytokine, IL-6, was positively correlated with fear/anxiety (r = .71, p = 0.03) and anger/physical aggression (r = .69, p = 0.04). IL-6 was marginally correlated with perceived stress (r = .66, p = 0.05).

**Conclusions**

Although both the NFL and MMA groups experience repetitive blows to the head, neither group demonstrated cognitive impairments relative to a control group. The NFL group showed significant impairments on three measures of negative affect (fear/anxiety, perceptions of physical anxiety, and anger/physical aggression) and on the measure of perceived stress.

## A6 Practice in REM: Motor Skill Consolidation During Sleep

### Kayla Thompson^1^, Michelle Filicko^1^, Jose Antonio^2^, Cheng Qian^3^, Jonathan B. Banks^1^, Jaime L. Tartar^1^

#### ^1^Department of Psychology and Neuroscience, Nova Southeastern University, Ft. Lauderdale, FL USA; ^2^Department of Health and Human Performance, Nova Southeastern University, Ft. Lauderdale, FL USA; ^3^Enchanted Wave LLC, Ft. Lauderdale, FL USA

##### **Correspondence:** Jaime L. Tartar (tartar@nova.edu)

**Background**

Sleep plays a critical role in the consolidation of memory and REM sleep has been particularly implicated in the consolidation of procedural memory (i.e., learned movement sequences). Procedural memory consolidation, not only stabilizes a memory, but also enhances performance. Despite the strong association between REM sleep and procedural memory consolidation, there fails to be a consensus in the literature regarding the timing and amount of REM sleep required for procedural memory consolidation. Thus, we tested the hypothesis that procedural memory is consolidated during REM sleep.

**Materials and Methods**

Participants were tested in a 4-day testing period using a validated 5-digit span finger tapping task. All subjects were provided a Single channel EEG headband (Enchanted Wave) and a wrist actigraphy device (Phillips Respironics) to wear for three consecutive nights (4 days) to record sleep data. The first two nights consisted of baseline measures of sleep. Following the 2^nd^ night of sleep (i.e. the 3^rd^ day), subjects were given the initial finger tapping “learning” task. After a subsequent night of sleep (4^th^ day), the participants were retested with the “post training” and “generalized-untrained-post” finger tapping task.

**Results**

Preliminary results show an increase in speed and accuracy of the finger tapping task after a subsequent night of sleep. Additionally, speed and accuracy were shown to increase in the “generalized-untrained-post” task, indicating that there is general consolidation of the motor skills that is not specific to the exact same sequence.

**Conclusions**

In this EEG-based sleep study to evaluate procedural memory consolidation during REM sleep, we show that sequential motor skills are enhanced after a night of sleep. Furthermore, REM sleep provides a novel neural environment to train procedural skill learning. These results can lead to substantial performance benefits in athletes and novel sleep-based training paradigms by optimally increasing athlete performance with a regimen consistent of training followed by sleep.

## A7 The effects of BANG® energy drink on psychomotor vigilance

### Christopher Horn^1^, Madaline Kenyon^1^, Cassandra Carson^1^, Anya Ellerbroek^1^, Lia Jiannine^1^, Tobin Silver^1^, Corey Peacock^1^, Jaime Tartar^2^, Jose Antonio^1^

#### ^1^Department of Health and Human Performance, NSU Florida, Davie, FL USA; ^2^Department of Psychology and Neuroscience, NSU Florida, Davie, FL USA

##### **Correspondence:** Jose Antonio (ja839@nova.edu)

Energy drinks, which contain caffeine as its primary active ingredient, have been shown to have an ergogenic effect vis a vis various athletic activities. However, it is not entirely known if energy drinks have an effect on psychomotor vigilance. The psychomotor vigilance test is a tool that is used to measure one’s behavioral alertness. It is a visual test that involves measuring the speed at which a person reacts to visual stimuli (e.g. a red dot against a black background) over a fixed time frame (e.g., 5 minutes).

**Materials and Methods**

Twenty exercise-trained men (n=11) and women (n=9) (mean±SD: age 32±7 years; height 169±10 centimeters; weight; 74.5±14.5 kilograms; % body fat 20.3±6.2 %; years of training 14±9; daily caffeine intake 463±510 milligrams) volunteered for this randomized, double-blind, placebo-controlled crossover trial. Body composition was determined via bioelectrical impedance (InBody 770). Subjects came to the lab twice with a washout period of ~7 days between testing. In a randomized order, they consumed either the BANG energy drink or a similar tasting placebo drink. In the second visit, they consumed the alternate drink. Thirty minutes post-consumption, they performed the following tests in this order: motor praxis test, psychomotor vigilance test, three sets of push-ups, motor praxis test, and psychomotor vigilance test. Reaction time (i.e., mean, fastest, slowest) was recorded. For the psychomotor vigilance test, lapses, false starts and efficiency score as also assessed.

**Results**

There were no differences between groups (BANG energy drink versus placebo) for any measure related to the motor praxis test. However, the BANG energy drink treatment resulted in a significantly lower (i.e., faster) psychomotor vigilance mean reaction time compared to the placebo (p=0.0220) (BANG energy drink 473.8±42.0 milliseconds, Placebo 482.4±54.0 milliseconds). Furthermore, the BANG energy drink treatment also produced a significantly higher (p=0.0250) efficiency score than the placebo (BANG energy drink 723.4±207.1; Placebo 660.4±223.4). Also, the BANG energy drink condition resulted in a lower number of lapses (i.e., reaction time > 500 milliseconds) (p=0.0608).

**Conclusions**

The acute consumption of BANG energy drink produced a significant improvement in psychomotor vigilance in exercise-trained men and women. In addition, efficiency was improved and the number of lapses diminished.

**Acknowledgements**

The product and placebo were provided by VPX.

## A8 Assessment of the FTO gene polymorphisms in exercise-trained men and women: the effects of a 4-week hypocaloric higher protein diet on body composition, part I

### Madaline Kenyon^1^, Sarah Knafo^2^, Alina Ali^2^, Cassandra Carson^1^, Anya Ellerbroek^1^, Cailey Weaver^2^, Lia Jiannine^1^, Tobin Silver^1^, Corey Peacock^1^, Jaime Tartar^2^, Jose Antonio^1^

#### ^1^Department of Health and Human Performance, NSU Florida, Davie, FL USA; ^2^Department of Psychology and Neuroscience, NSU Florida, Davie, FL USA

##### **Correspondence:** Jose Antonio (ja839@nova.edu)

**Background**

Variations in the fat mass and obesity-associated gene (FTO) are associated with obesity; however, it is not clear if changes in energy intake affect the adaptive response to caloric restriction in those with the risk variants. The three FTO single nucleotide polymorphisms (SNPs), rs1421085, rs17817449 and rs9939609, are in strong linkage disequilibrium. Thus, the purpose of this investigation was to determine the role of the FTO gene vis-à-vis the effects of a 4-week hypocaloric diet on body composition in exercise-trained men and women. We also assessed several biomarkers derived from a saliva sample (i.e., cortisol, C-reactive protein, alpha-amylase).

**Materials and Methods**

Forty-seven exercise-trained men (n=11) and women (n=36) (mean±SD: age 32±9 years; height 169±8 centimeters, body mass index 24.5±2.9, hours of aerobic training per week 4.9±3.8, hours of weight training per week 3.9±2.4, years of training experience 13.4±7.0) completed a 4-week hypocaloric diet (i.e., decrease total calories by ~20% while maintaining a protein intake of ~2.1 g/kg/d). Subjects were instructed to maintain the same training regimen and to decrease energy intake via carbohydrate and/or fat restriction during the treatment period. Body composition was determined pre and post via dual energy x-ray absorptiometry (DXA). Total body water was determined via a multifrequency bioelectrical impedance device (InBody 770). Saliva samples were collected pre and post in order to genotype the participants as well as estimate the concentration of several biomarkers (see part II). All testing was done between 11:30am and 3pm.

**Results**

Of the 47 subjects, 15 were of normal risk for obesity whereas 32 were carriers of the risk alleles for the FTO gene. Subjects were grouped based on their genotype for the three FTO SNPs (i.e., rs1421085, rs17817449 and rs9939609) due to their strong linkage disequilibrium. We have classified those with the normal obesity risk as “non-risk allele” versus those that carry the “risk allele” (i.e., both heterozygous and homozygous). Both groups experienced a significant decrease in total energy intake (non-risk allele: pre kcal 2081±618, post kcal 1703±495; risk allele: pre kcal 1886±515, post kcal 1502±366). Both groups lost a significant amount of body weight (risk allele change: -1.0±1.2, non-risk allele change: -1.2±1.4) and fat mass (risk allele change: -1.1±0.7, non-risk allele change: -0.9±0.4) with no significant differences between the groups. There were no significant changes in either group for fat free mass or total body water.

**Conclusions**

In the short-term (i.e., four weeks), exercise-trained men and women consuming a hypocaloric diet that is relatively high in protein experience similar changes in body composition due exclusively to a decrement in fat mass. Furthermore, the changes are similar whether you are at a normal risk or are at higher risk for obesity based on their FTO genotypes.

## A9 Assessment of the FTO gene polymorphisms in exercise-trained men and women: the effects of a 4-week hypocaloric higher protein diet on salivary cortisol and alpha-amylase, part II

### Jose Antonio^1^, Sarah Knafo^2^, Madaline Kenyon1, Alina Ali^2^, Cassandra Carson^1^, Anya Ellerbroek^1^, Cailey Weaver^2^, Corey Peacock^1^, Jaime Tartar^2^

#### ^1^Department of Health and Human Performance, NSU Florida, Davie, FL USA; ^2^Department of Psychology and Neuroscience, NSU Florida, Davie, FL USA

##### **Correspondence:** Jose Antonio (ja839@nova.edu)

**Background**

Variations in the fat mass and obesity-associated gene (FTO) are associated with obesity; however, it is not clear if changes in energy intake affect the adaptive response to caloric restriction in those with the risk variants. The three FTO single nucleotide polymorphisms (SNPs), rs1421085, rs17817449 and rs9939609, are in strong linkage disequilibrium. Thus, the purpose of this investigation was to determine the role of the FTO gene vis-à-vis the effects of a 4-week hypocaloric diet on body composition in exercise-trained men and women. We also assessed several biomarkers (i.e., salivary alpha-amylase – a marker of sympathetic nervous system activity; salivary cortisol – a biomarker of psychological stress).

**Methods**

Forty-seven exercise-trained men (n=11) and women (n=36) (mean±SD: age 32±9 years; height 169±8 centimeters, body mass index 24.5±2.9, hours of aerobic training per week 4.9±3.8, hours of weight training per week 3.9±2.4, years of training experience 13.4±7.0) completed a 4-week hypocaloric diet (i.e., decrease total calories by ~20% while maintaining a protein intake of ~2.1 g/kg/d). Subjects were instructed to maintain the same training regimen and to decrease energy intake via carbohydrate and/or fat restriction during the treatment period. Body composition was determined pre and post via dual energy x-ray absorptiometry (DXA). Total body water was determined via a multifrequency bioelectrical impedance device (InBody 770). Saliva samples were collected pre and post in order to genotype the participants as well as estimate the concentration of several biomarkers. All testing was conducted between 11:30am and 3pm.

**Results**

Of the 47 subjects, 15 were of normal risk for obesity whereas 32 were carriers of the risk alleles for the FTO gene. Subjects were grouped based on their genotype for the three FTO SNPs (i.e., rs1421085, rs17817449 and rs9939609) due to their strong linkage disequilibrium. We have classified those with the normal obesity risk as “non-risk allele” versus those that carry the “risk allele” (i.e., both heterozygous and homozygous). Both groups experienced a significant decrease in total energy intake and lost a significant amount of body weight and fat mass. There were no differences in salivary alpha-amylase or cortisol.

**Conclusions**

There is no difference between individuals in the risk allele vs. non-risk allele groups with regards to the “stress response” vis-à-vis salivary alpha-amylase or cortisol (i.e., FTO gene SNPs: rs1421085, rs17817449 and rs9939609).

## A10 Body composition assessment: DXA, Bod Pod, and InBody comparison

### Anya Ellerbroek^1^, Madaline Kenyon1, Cassandra Carson^1^, Denvyr Tyler-Palmer^1^, Tobin Silver^1^, Jaime Tartar ^2^, Sarah Knafo^2^, Victoria Burgess^1^, Lia Jiannine,^1^ , Victoria Burgess^1^, Corey Peacock^1^ , Jose Antonio^1^

#### ^1^Exercise and Sport Science, NSU Florida, Davie, FL USA; ^2^Department of Neuroscience, NSU Florida, Davie, FL USA

##### **Correspondence:** Jose Antonio (ja839@nova.edu)

**Background**

There are multiple laboratory methods for assessing body composition that are utilized. Each method has its advantages and disadvantages. The purpose of this investigation was to compare the body composition measures in a cohort of 155 exercise-trained men and women.

**Materials and Methods**

Body composition was assessed in a cohort of exercise-trained men and women via air displacement plethysmography (Bod Pod®), dual energy x-ray absorptiometry (DXA) and a multifrequency bioelectrical impedance device (InBody 770®). Subjects (n=155; 42 male, 113 female) came to the laboratory for body composition assessment (i.e., percent body fat, lean body mass and fat mass) between 11:00am and 3:00pm. Subjects were instructed to not eat three hours prior to testing and avoid exercise the morning of.

**Results**

One hundred and fifty-five subjects volunteered for this investigation (n=155; n=113 female, n=42 male; Age 27±9 years, Height 170±10 centimeters, Weight 71.4±14.7 kilograms). All subjects were self-reported to be exercise-trained. There were no significant differences between the Bod Pod and InBody for any measure (i.e., fat mass, lean body mass, percent body fat); however, the DXA had significantly higher percent fat (p<0.0001) compared to the Bod Pod and InBody (mean±SD for % body fat for male and female subjects combined: Bod Pod 21.3±8.8, DXA 25.9±7.6 InBody 21.8±9.2). For female subjects, there were no differences for any measure between the InBody and Bod Pod; however, the InBody and Bod Pod underpredicted fat mass and overpredicted lean body mass in comparison to the DXA. On the other hand, There was no difference between the Inbody, Bod Pod and DXA for measures of lean body mass and fat mass in male subjects.

**Conclusions**

There were no significant differences between the Bod Pod and InBody for any measure of body composition. However, both the Bod Pod and InBody overestimated lean body mass and underestimated fat mass and percent body fat in comparison to the DXA in female subjects.

## A11 Comparison of Dual-Energy X-Ray Absorptiometry (DXA) versus Multifrequency Bioelectrical Impedance (InBody 770) for Body Composition Assessment After a 4-Week Hypoenergetic Diet

### Cassandra Carson^1^, Madaline Kenyon^1^, Anya Ellerbroek^1^, Denvyr Tyler-Palmer^1^, Tobin Silver^1^, Jaime Tartar ^2^, Victoria Burgess ^1^, Sarah Knafo^2^, Lia Jiannine^1^, Jonathan Mike^3^, Corey Peacock^1^, Jose Antonio^1^

#### ^1^Exercise and Sport Science, NSU Florida, Davie, FL USA; ^2^Department of Neuroscience, NSU Florida, Davie, FL USA; 3Exercise Science, Grand Canyon University, Phoenix AZ USA

##### **Correspondence:** Jose Antonio (ja839@nova.edu)

**Background**

A plethora of studies have compared body composition estimates from the multifrequency BIA device used in this study, the DXA and the Bod Pod (air displacement plethysmography). Because body composition may be important in health and and performance and is often critical during physique competitions utilizing a caloric deficit, the purpose of this study was to compare the use of dual-energy x-Ray absorptiometry (DXA) versus multifrequency bioelectrical impedance (InBody 770) for body composition assessment after a 4-week hypoenergetic diet.

**Materials and Methods**

Subjects were instructed to reduce their energy intake by 25% during the treatment period. Our pool of subjects typically logged their food on a regular basis. Thus, they were well versed at tracking their intake. Subjects were also instructed initially to provide a baseline food log prior to testing (24-hour recall); subsequently, they were instructed to keep a food log (three times per week) on the MyFitnessPal mobile app for the duration of the treatment period. Body composition was assessed with a dual-energy X-ray absorptiometry machine (DXA) (Model: Hologic Horizon W; Hologic Inc., Danbury CT USA) and an InBody 770 multifrequency bioelectrical impedance (BIA) device. Subjects were instructed to come to the laboratory after at least a 3-hour fast and no prior exercise that day. All testing was performed between 11:00am and 3:00pm.

**Results**

On average, subjects (n=41 [29 female, 12 male]; mean±SD: age 33±10 years, height 169±8 centimeters, average years of exercise training 14±6 years) reduced their energy intake by 17.1±11.2 percent. The BIA underestimated fat mass and percentage body fat and overestimated lean body mass in comparison to the DXA. However, when assessing the change in fat mass, lean body mass or percent body fat, there were no statistically significant differences between the BIA vs. DXA. Overall, the change in percent body fat using the DXA vs. the Inbody was -1.3±0.9 and -1.4±1.8, respectively.

**Conclusions**

Our data suggest that when tracking body composition over a period of four weeks, the multifrequency BIA (Inbody 770) may be a viable alternative to the DXA in exercise-trained men and women.

## A12 The effects of maca on grip strength, fatigue, and sexual behavior in men and women

### Lia Jiannine, Jose Antonio

#### Department of Health and Human Performance, NSU Florida, Davie, FL USA

##### **Correspondence:** Lia Jiannine (LJiannine@nova.edu)

**Background**

Domesticated maca has been grown in Peru for over 2,000 years and is a staple crop for natives of Peru. There are a variety of medicinal claims regarding the efficacy of maca, including improvements in fertility, sexual functioning, energy, stamina, and physical performance.

**Materials and Methods**

This study used a randomized, double-blind, placebo-controlled design that examined the effect of maca on outcome measures such as body composition, grip strength, mood, and sexual functioning. Forty-seven subjects (ages 18 to 53) were randomized into either treatment (25 subjects) or placebo group (22 subjects). Participants were instructed to ingest 2.1g of a maca-containing product (Nutrition21 proprietary maca blend - Lepidamax^TM^) or placebo each day for 28-days. Both maca and placebo were divided into three pills.

Participants were required to visit the body composition lab on two separate occasions for pre-and post-testing. Anthropometric measurements consisted of height, weight, and body fat percentage. Grip strength was tested in the dominate hand through the Jamar hand grip device. On study visit days, participants also filled out two questionnaires. The first was the Profile of Mood States (POMS), a 65-item multidimensional physiological rating scale used to assess transitory mood states including Tension, Depression, Anger, Vigor, Fatigue, and Confusion. The second survey was adapted from the Derogates Interview for Sexual Functioning (DISF- SR), which contains 26 questions separated into five domains: Sexual Cognition/Fantasy, Sexual Arousal, Sexual Behavior and Experience, Orgasm and Sexual Drive.

Data were analyzed using SPSS software. All dependent variables were tested using the Shapiro test. The Nonparametric Wilcoxon rank-sums test was utilized to examine differences between the groups for men and women separately, based on the small sample size and the distribution of the data. Differences were considered significant for the one-tailed *P*-value < 0.05.

**Results**

In men, maca significantly improved handgrip strength (p = 0.0371), sexual behavior (p = 0.0185) and fatigue (p = 0.0456) when compared to placebo. Although females in the treatment group had significant changes in fatigue, confusion, tension, orgasm and handgrip from baseline while placebo did not (p < 0.05), those differences were not significant when compared to the placebo group.

**Conclusions**

Study results showed that maca lessened fatigue, improved strength and enhanced sexual functioning in men and women, with efficacy more pronounced in men. These effects may have displayed greater significance in women with a larger study sample. The results of this study support the use of maca to improve male performance.

## A13 NFL Combine Training: Body Composition Assessment

### Pathik Vaidya, Monique Mokha, Pete Bommarito, Jose Antonio, Lia Jiannine

#### Department of Health and Human Performance, NSU Florida, Davie, FL USA

##### **Correspondence:** Jose Antonio (ja839@nova.edu)

**Background**

Prior to the NFL combine, players partake in NFL training camps to improve their health and athletic ability in preparation for their professional debut. Players attempt to improve performance in various tests such as the vertical jump, broad jump, 40-yard dash and 3-cone drill, as well as, their body composition. The NFL has used the BodPod for the last decade to measure body composition at the combine. The BodPod measures the volume of air displaced by an individual and uses algorithms and the individual’s height and weight in order to determine the amount of percent body fat (%BF) and fat free mass.

**Materials and Methods**

Various collegiate football players training for the NFL combine volunteered for this study. Their body composition was measured using both volumetric air displacement (BodPod) and a multifrequency bioelectrical impedance device (InBody 770). Athletes were tested twice, once prior to attending the training program, and once afterwards.

**Results**

There were no significant differences in body composition from pre- and post- training camp testing. The average fat mass of athletes was slightly lower in post testing than pre-testing. However, this difference was not statistically significant.

**Conclusions**

Body composition did not change significantly as a result of NFL training program.

## A14 Body composition and grip strength: an investigation of the relationship in change scores.

### Tatyana Salguero, Todd Chou, Monique Mokha, Pete Bommarito, Jose Antonio, Lia Jiannine

#### Department of Health and Human Performance, NSU Florida, Davie, FL USA

##### **Correspondence:** Jose Antonio (ja839@nova.edu)

**Background**

A player has a better chance of being recruited during the NFL Combine after participating in a training program designed to better each athlete’s health and athletic skills. Grip strength may play a role in how well one is able to perform. The following study researches whether an NFL training program has an effect on an individual's grip strength and if changes in body composition were related to changes in grip strength

**Materials and Methods**

Nine collegiate football players training for the NFL combine voluntarily participated in this study. Each players grip strength was measured using an Analog Baseline® Dynamometer. Two recording were taken for each player’s dominant hand. Body composition was measured using two methods. The first one involved the use of air displacement (BodPod) and the second method calculated body composition through bioelectrical impedance analysis (InBody 770). Each player had their grip strength and body composition measured twice. They were measured before beginning their training program and after completing the program.

**Results**

There were no significant differences in grip strength. Players had an average grip strength of 61.1 kg at pre-test and 57.9 at post-test. There were also no significant changes in body composition. Mean body composition was 13.1% and pre-test and 13.4% at post-test. Change in body composition was unrelated to change in grip strength.

**Conclusions**

Changes in both grip strength and body composition were not significant and there was no relationship between the change score of the two variables.

## A15 Allele Frequencies of the “Warrior/ Worrier” COMT Polymorphism in MMA fighters, Athletes, and non-Athletes

### Corey A. Peacock^1^, Sarah Knafo^2^, Valeria Nazaire^3^, Dominick Cabrera^2^, Julius D. Thomas^2^, Jose Antonio^1^ Jaime L. Tartar^1^

#### ^1^Nova Southeastern University, College of Health Care Sciences, Ft. Lauderdale, FL USA; ^2^Nova Southeastern University, College of Psychology, Ft. Lauderdale, FL USA; ^3^Miami Dade College, College of Science, Miami, FL USA

##### **Correspondence:** Jaime L. Tartar (tartar@nova.edu)

**Background**

A functional single-nucleotide polymorphism (SNP) in the catechol-O-methyltransferase (COMT) gene (rs4680) holds great promise as a gene variant that can predict the ability to maintain cognitive agility (e.g. sustained attention and processing speed) during combat and competition. The COMT enzyme works to catabolize catecholamines in the central and peripheral nervous systems. The COMT (Val158Met) SNP results in a twofold to fourfold decrease in the activity of the COMT enzyme and increase in dopamine levels- especially in the pre-frontal cortex (PFC). Critically, the COMT Met (low-activity; high dopamine) allele carriers outperform Val (high-activity; low dopamine) homozygotes on a variety of cognitive tasks. However, this relationship between genotype and cognitive performance appears to reverse under stressful conditions. Stress increases PFC DA levels, and Met allele carriers (with higher DA) show performance deficits relative to Val allele carriers. This pattern reflects the inverted U-shaped function of DA activity where too little (Val allele) or too much (Met allele carriers under stress) DA is associated with poor cognitive performance. The Val allele advantage for stress resiliency is referred to as the COMT “warrior/ worrier” model. In line with this model, we predicted that elite, UFC level, MMA fighters would be more likely than athlete controls to carry the GG (warrior) genotype and that both groups would have a higher GG allele frequency relative to non-athlete controls.

**Materials and Methods**

19 MMA fighters (mean age = 30.19, SD = 5.16), 21 non-contact sport athlete controls (M age = 31.19, SD = 12.16), and 41 non-athlete controls (mean age = 22.66, SD = 4.14) were genotyped for the COMT 4680 polymorphism. The study only included male participants. Genomic DNA was extracted in a QIAcube instrument following the manufacturer’s standard protocol for saliva nucleic acid extraction (QIAGEN, Valencia, CA, USA). After isolation, allelic discrimination for the COMT gene was determined via real-time polymerase chain reaction (PCR) using a TaqMan SNP genotyping assay using fluorogenic probes (Applied Biosystems, CA, USA). Genotypes were determined automatically via the StepOne software (Applied Biosystems) based on the fluorescence signals. Samples were run in duplicate and in the case of a call discrepancy, samples were rerun.

**Results**

We carried out a series of Chi Square analyses. A 3 (group) X 2 (COMT) analyses showed an overall significant difference in genotype frequencies between groups X^2^ =9.32, p = 0.01. There was also a statistically significant difference between the MMA group and the non-athlete control group X^2^ =8.84, p = 0.002. However, there was not a statistically significant difference between the athlete control group and the non-athlete control group X^2^ =3.80, p = 0.05. There was also not a statistically significant difference between the MMA group and the athlete control group X^2^ =0.90, p = 0.34.

**Conclusions**

Our data show a trend for an increase in the warrior (GG) genotype wherein MMA fighters have the highest frequency (58%) followed by the athlete control group (43%) and the non-athlete control group showing the lowest frequency (20%). Combined, our findings suggest that The “Warrior” genotype may play a role in participation in competitive sports, and especially combat sports.

**Support**

This study was supported by a Department of Education sub-contract award to JLT (P031C160143)

## A16 The Acute Effects of Citrulline Malate and Bonded Arginine Silicate Supplementation on Vasodilation of Young Adults

### Jeffrey M. Rogers, Josh Gills, Michelle Gray

#### Exercise Science Research Center, University of Arkansas, 1 University of Arkansas, HPER 321-E, Fayetteville, AR 72701, USA

##### **Correspondence:** Jeffrey M. Rogers (rogersdst@gmail.com)

**Background**

Clinicians, professional athletes, and recreational athletes are interested in supplementation that up-regulates nitric oxide (NO) production in blood vessel endothelium, increasing arterial vasodilation. Benefits from these supplements include improvements in blood pressure, muscle hyperemia, and exercise performance. Citrulline Malate (CM) is a pre-workout ingredient, popular for its ability to increase exercise performance and blood serum concentrations of L-arginine, resulting in NO production. Recently, Inositol-Stabilized Arginine Silicate (ASI, Nitrosigine) has been added to many of the most popular pre-workout blends, following a group of studies showing ASI increases serum arginine and reduces post-workout muscle damage. Research has yet to compare CM and ASI in-vivo using a flow-mediated dilation (FMD) technique, a validated measure of the vascular endothelium’s NO producing ability. Thus, the purpose of this experiment was to determine the effectiveness of ASI, compared to CM and placebo, in up-regulating NO production in blood vessels as measured by acute changes in vasodilation.

**Method and Materials**

We utilized a double-blind, within-subjects design where participants reported for three trials, each preceded by a 7-day washout period. Upon reporting to the research center, participants read and signed the informed consent document, gave a brief medical history, and remained in an upright-seated position until their blood pressure and heart rate normalized. The participant then reclined into a comfortable supine position in a phlebotomy chair, and his or her arm was abducted at 70 to 90 degrees and at heart level, depending on the participant’s level of comfort. A baseline FMD measurement was obtained followed by consumption of one clinical dose CM (8g), ASI (1.5g), or dextrose placebo (8g); the supplementation order was randomized controlling for potential order effects. Participants completed a brief 24-hour nutrition survey and waited for 60 minutes. After the waiting period, FMD was repeated. We used screen capture software to record the entire FMD procedure and conducted analyses on the videos using Quipu Cardiac Suite software.

**Results**

Repeated measures analysis of variance yielded a significant supplement x time effect (*p*<.001), such that CM and ASI yielded a greater change in FMD response than placebo. After allometric scaling of the FMD values, supplement x time effect remained significant (*p*=.001).

**Conclusions**

Both CM and ASI may be particularly beneficial to individuals looking to increase the potential for muscle hyperemia during exercise. Our results support previous findings that CM and ASI increase blood serum concentrations of arginine, and are effective at increasing vascular endothelium nitric oxide producing capacity.

**Acknowledgements**

We conducted this study with funding provided by a State Undergraduate Research Fund grant and the University of Arkansas Honors College.

## A17 A comparison between recommended kilocalorie and macronutrient intake and reported intake of collegiate women’s soccer players

### Shelley L. Holden and Neil A. Schwarz

#### University of South Alabama, Mobile, AL, USA

##### **Correspondence:** Shelley L. Holden (sholden@southalabama.edu)

**Background**

The purpose of this study was to compare the International Society of Sports Nutrition (ISSN)

recommendations for daily macronutrient and kilocalorie (kcal) intake to reported intake of

collegiate female soccer players.

**Materials and Methods**

Twenty NCAA Division I female soccer players (162.18 ± 9.75 cm; 65.96 ± 18.5 kg; 18.52 ±

2.27 % body fat, 22.5 ± 2.07 kg/m 2 ) self-reported dietary intake during the first three days of the pre-season and for four days during mid-season competition. Participants bodyweight was used to calculate recommended ISSN macronutrient and energy values. Average daily total energy and macronutrient intake were calculated separately for the pre-season and mid-season periods. Reported intakes were compared to nutritional recommendations set forth by the ISSN for team sport athletes (50 kcal/kg/d with 30% fat; 6.5 g/kg/d carbohydrate; and 1.8 g/kg/d protein). Data were analyzed using repeated-measures ANOVA with an a priori alpha level of p = 0.05. Tukey’s post-hoc tests were used for multiple comparisons.

**Results**

Reported energy intake for mid-season (1621 ± 424 kcal/d) was significantly lower (p < 0.01)

than pre-season (2082 ± 494 kcal/d) and both were significantly less (p < 0.01) than the ISSN

recommendation (3095 ± 368 kcal/d). Reported carbohydrate intake for mid-season (191 ± 54

g/d) was significantly lower (p < 0.01) than pre-season (252± 69 g/d) and both were significantly less (p < 0.01) than the ISSN recommendation (404 ± 45 g/d). Reported fat intake was similar (p = 0.08) between pre-season (72 ± 26 g/d) and mid-season (57 ± 24 g/d), but both were significantly lower (p < 0.01) than the ISSN recommendation (103.6 ± 11.5 g/d). Reported

protein intake for mid-season (69 ± 25 g/d) was significantly lower (p < 0.01) than reported

intake for pre-season (101 ± 27 g/d) and ISSN recommendations (112 ± 12 g/d). Reported

pre-season protein intake was equivalent to the ISSN recommendation ( p = 0.25).

**Conclusions**

Overall, reported energy and macronutrient intake of NCAA Division I female soccer players is

lower than the recommendations set forth by the ISSN. Future research should explore the

accuracy of self-reported data in this population and if nutrition education and/or interventions

can help them meet recommendations and improve recovery and performance.

## A18 Dietary supplementation practices in female Division II athletes

### Danielle Pope, Lorena Hernandez, Jose Antonio, Lia Jiannine

#### Nova Southeastern University, Davie, FL, USA

##### **Correspondence:** Lia Jiannine (ljiannine@nova.edu)

**Background**

Sport supplements aid in recovery and athletic performance as well as improve overall health. Ergogenic aids consist of vitamins, minerals, amino acids or herbs that aid in performance and are available over the counter. This study investigated the types of supplements, frequency of supplement use, and reasons for supplementation.

**Materials and Methods**

Ninety-one Division II female athletes anonymously responded to a google questionnaire. Participants provided demographic information and listed their collegiate sport.

Athletes completed a comprehensive survey detailing supplementation patterns with respect to frequency and reasons for use.

**Results**

Out of 91 responses, 75.8% take sport supplements. Of those that take supplements, 82.4% reported taking supplements for sport recovery, 48.5% for performance, and 26.5% for muscle gain. Protein was the most commonly used supplement; 66.7% percent used whey powder, followed by protein bars (62.1%). Surprisingly, only 1 out of 69 participants added vitamin C, Biotin, Iron, and/or a women’s vitamin into their diets and none of these athletes added beta-alanine. When athletes reported reasons for supplementation, 40.9% of participants indicated that they were recommended by the coaching staff, 37.9% recommended by a teammate, 31.8% recommended by family, and 22.7% indicated that they take supplements recommended by a physician and/or nutritionist.

**Conclusions**

Over 75% of the Division II athletes surveyed consumed sports supplements. Since the majority of athletes take supplements for recovery, it is unsurprising that whey protein was the most commonly used supplement. The majority of athletes took supplements daily. Athletes listed recommendations by coaching staff followed by recommendations by teammates as the two most common external motivating factors.

## A19 Caffeine timing improves lower-body muscular performance

### Patrick S. Harty^1^, Hannah A. Zabriskie^1^, Richard A. Stecker^1^, Bradley S. Currier^1^, Andrew R. Jagim^2^, Scott R. Richmond^1^, Cynthia A. Schroeder^1^, Chad M. Kerksick, FISSN^1^

#### ^1^Exercise and Performance Nutrition Laboratory, School of Health Sciences, Lindenwood University, St. Charles, MO, USA; ^2^Human Performance Lab, Division of Family Medicine, Mayo Clinic Health System, Onalaska, WI, USA.

##### **Correspondence:** Chad M. Kerksick (ckerksick@lindenwood.edu)

**Background**

Caffeine is a commonly-consumed stimulant that has been shown to improve force production, power production, and muscular endurance in a variety of populations. However, little is known about the optimal time to consume caffeine prior to exercise to maximize the ergogenic benefits of the substance. The purpose of this investigation was to determine the optimal pre-exercise time interval to consume caffeine to maximize force production, fatigue resistance, and power production. Secondary purposes of this investigation were to determine the presence of any gender differences in responses to timed caffeine administration and identify the general ergogenic effect of acute caffeine supplementation on muscular performance.

**Materials and Methods**

Healthy, resistance-trained males (*n*=18; Mean±SD; Age: 25.1±5.7 years; Height: 178.4±7.1 cm; Body mass: 91.3±13.5 kg; Percent bodyfat: 20.7±5.2; Average caffeine consumption: 146.6±100.3 mg·day^-1^) and females (*n*=11; Mean±SD; Age: 20.1±1.6 years; Height: 165.0±8.8 cm; Body mass: 65.8±10.0 kg; Percent bodyfat: 25.8±4.2; Average caffeine consumption: 111.8±91.7 mg·day^-1^) were recruited to participate in this investigation. In a randomized, double-blind, placebo-controlled, crossover fashion, participants consumed 6 mg·kg^-1^ caffeine or placebo solution at three time points: 2 hours prior (2H), 1 hour prior (1H), or 30 minutes prior (30M) to exercise testing. During 3 visits, caffeine was administered at one time point, while placebo was administered at all time points during one visit. Following consumption of the supplements, participants performed a lower-body testing battery consisting of isometric mid-thigh pulls (IMTP), countermovement vertical jumps (CMVJ), and isometric/isokinetic knee extensor testing (ISO/ISOK). Repeated measures ANOVAs, paired-samples t-tests, and effect size calculations were used to analyze all outcomes.

**Results**

Caffeine administered at 1H significantly improved absolute CMVJ and ISO performance relative to placebo. Mean CMVJ jump height was significantly higher during 1H compared to 30M. However, only caffeine administered at 30M significantly improved absolute measures of isokinetic performance. Analysis of the pooled caffeine conditions and the change in performance for each condition relative to placebo revealed that muscular performance was more consistently augmented by caffeine in males compared to females.

**Conclusions**

Pre-exercise caffeine timing significantly modulated responses to the substance, with 1H exerting the most consistent ergogenic benefits relative to other time points, particularly compared to 2H. Male participants were found to respond more consistently to caffeine compared to female participants. These results suggest that active individuals can maximize the ergogenic effects of caffeine by consuming the substance approximately one hour prior to the point when peak muscular performance is desired.

**Acknowledgments**

The authors would like to thank all participants for their involvement in the study. This project was funded by the National Strength and Conditioning Association Foundation. The authors declare no conflicts of interest.

## A20 Skeletal muscle sarcoplasmic and myofibrillar proteomic comparisons between younger resistance-trained versus untrained younger and older humans

### Christopher G. Vann^1^, Paul. A. Roberson^1^, Shelby C. Osburn^1^, Petey W. Mumford^1^, Matthew A. Romero^1^, Carlton D. Fox^1^, Johnathon H. Moore^1^, Cody T. Haun^2^, Andreas N. Kavazis^1,4^, Veera L.D. Badisa^3^, Benjamin M. Mwashote^3^, Victor M. Ibeanusi^3^, Darren T. Beck^1,4^, Jordan R. Moon^5^, Kaelin C. Young^1,4^, Michael D. Roberts^1,4^

#### ^1^School of Kinesiology, Auburn University, Auburn, AL, USA; ^2^Department of Exercise Science, LaGrange College, LaGrange, GA, USA; ^3^School of the Environment, Florida A&M University, Tallahassee, FL, USA; ^4^Department of Cell Biology and Physiology, Edward Via College of Osteopathic Medicine - Auburn Campus, Auburn, AL, USA; ^5^ImpediMed Inc., Carlsbad, CA, USA

##### **Correspondence:** Michael D. Roberts (mdr0024@auburn.edu)

**Background**

Evidence suggests that aging, and paradoxically longer-term resistance training, reduces skeletal muscle myofibrillar protein concentrations or volume. While the former occurrence is an age-related phenotype, the latter may be due to a phenomenon termed “sarcoplasmic hypertrophy” wherein increases in sarcoplasmic protein concentrations and/or volume predate the accretion of myofibrillar proteins. The purpose of this study was to examine the myofibrillar and sarcoplasmic proteomes of skeletal muscle biopsies obtained from younger resistance-trained (YT, n=6, 25±4 years old, 9.8±3.0 reported years of training) as well as healthy younger (YU, n=6, 21±1 years old) and older (OU, n=6, 62±8 years old) untrained individuals with the intent of identifying whether training or aging alters the relative abundances of said protein pools.

**Materials and Methods**

Participants performed a battery of tests and donated a vastus lateralis (VL) muscle biopsy in the morning hours following an overnight fast. VL sarcoplasmic and myofibrillar protein fractions were separated using differential centrifugation, protein concentrations were determined on each fraction, and actin and myosin concentrations were analyzed using SDS-PAGE.

**Results**

The following variables were different between cohorts (p<0.05): a) whole-body lean soft tissue mass (YT>YU&OU), b) VL thickness (YT>YU&OU), and leg extensor peak torque (YT>YU&OU). Myofibrillar protein concentrations were greater in YT versus OU (p=0.005) and trended higher in YU (p=0.091) versus OU, but were not different between the former two groups (p=0.325). Actin and myosin concentrations were greater in YT versus YU (p<0.05) and OU (p<0.001). Sarcoplasmic protein concentrations were not different between groups. When all participants were pooled, there were associations between VL thickness and myofibrillar protein concentration (r^2^=0.482, p=0.001) as well as myosin concentration (r^2^=0.441, p=0.004), but not between VL thickness and sarcoplasmic protein concentration (r^2^=0.084, p=0.241).

**Conclusions**

These data suggest VL myofibrillar protein concentrations decrease with aging. Additionally, long-term resistance training does not sustain sarcoplasmic hypertrophy, but instead may promote modest (~10%) myofibrillar protein packing.

## A21 Power and Velocity Parameters during the Sit to Stand in Young and Older Males

### Joseph B. Boone, Alex A. Olmos, Phuong L. Ha, Matthew T. Stratton, Alyssa R. Bailly, Micah J. Poisal, Joshua A. Jones, Benjamin E. Dalton, Tyler M. Smith, Trisha A. VanDusseldorp, Yuri Feito, Garrett M. Hester

#### Dept. of Exercise Science and Sport Management, Kennesaw State University, Kennesaw, GA, USA

##### **Correspondence:** Garrett M. Hester (ghester4@kennesaw.edu)

**Background**

It is well established that muscular power is important for physical function in older adults, however, velocity has only recently been identified as in important contributor to some functional tasks. Examining power and velocity simultaneously during a sit-to-stand (STS) may yield relevant insight regarding physical function in older adults. The purpose of this study was to compare power and velocity parameters during a single STS between young and older males.

**Materials and Methods**

Thirty healthy, untrained young (n=15; age=20.7 ± 2.2 yrs; BMI=23.1 ± 2.4 kg/m^2^;) and older (n=15; age=72.1 ± 4.0 yrs; BMI=29.6 ± 5.4 kg/m^2^) males were recruited for this study. Participants performed a single STS, which involved rising from a seated position to a standing position without assistance from the upper-body (arms crossed over their chest). A familiarization visit was completed prior to testing. Participants were instructed “to stand as quickly as possible” and three trials were performed. Knee joint angle was measured via manual goniometry between trials to ensure consistency across trials. Peak force, average power, average partial power, peak power, average velocity, and peak velocity were obtained during the STS using a Tendo Weightlifting Analyzer (Trencin, Slovak Republic) that was attached to the waist of participants. Body mass was entered in the Tendo microprocessor prior to each test. Peak force and average partial power were obtained from the first 50% of the movement, while all other variables were calculated using the full range of the movement. The trial with the highest average power was used for subsequent analysis. Independent samples t-tests were used to examine differences between groups.

**Results**

Peak power was decreased in older males (28%; p = 0.016), but peak force was similar between groups (p = 0.458). Average power was similar between groups (p=0.132), while partial average power was lower in older males (18%; p = 0.039). Peak and average velocity were decreased in older males (29%; p = 0.001 and 27%; p = 0.008, respectively).

**Conclusion**

These data suggest that there are preferential decrements in power and velocity parameters for older males during the STS. The age-related difference in velocity appears to be more dramatic than that of power considering the relative differences and the finding that average power was similar between groups. Relatedly, the finding that partial average power was lower in the older group, while peak force was similar indicates that a reduction in velocity was primarily influential for the decreased power, at least for the initial aspect of the STS. Improving velocity capacity may be of critical importance for interventions aimed at enhancing physical function in older adults.

## A22 Effect of Dynamine™ With and Without TeaCrine^®^ Over Four Weeks of Continuous Use on Cardiovascular Function and Psychometric Parameters of Healthy Males and Females

### Michaela G. Alesi^1^, Matthew T. Stratton^1^, Alyssa R. Bailly^1^ Alyssa J. Holmes^1^, Andrew Modjeski^1^, Megan Barie^1^, Yuri Feito^1^, Gerald T. Mangine^1^, Karleena R. Tuggle^2^, Tiffany A. Esmat^1^, Garrett M. Hester^1^, Katy Hayes^1^, and Trisha A. VanDusseldorp^1^

#### ^1^Dept. of Exercise Science and Sport Management, Kennesaw State University, Kennesaw, GA, USA; ^2^WellStar Medical Group Comprehensive Bariatric Services, Marietta, GA, USA

##### **Correspondence:** Trisha A. VanDusseldorp (tvanduss@kenensaw.edu)

**Background**

Methylliberine (1,7,9,tetramethlyluric acid, Dynamine™) and theacrine (Teacrine®) are pure alkaloids, naturally occurring in multiple species of *Coffea*, and are derived from caffeine. Previous research on Teacrine reported increases in feelings of energy, focus, and concentration, with a reduced sensation of fatigue. Currently there is no published human safety data available on Dynamine, despite our previously published pilot abstract. The purpose of this study was to examine the effects of four weeks of continuous use of Dynamine with and without TeaCrine on changes in heart rhythm (electrocardiogram; ECG), resting heart rate (RHR), blood pressure (BP), and psychometric parameters (PP).

**Materials and Methods**

One-hundred college aged men (n=43) and women (n=57) were randomly assigned to one of five groups: low dose Dynamine (100mg), high dose Dynamine (150mg), low dose Dynamine with TeaCrine (100mg Dynamine + 50mg TeaCrine), high dose Dynamine with TeaCrine (150mg Dynamine + 25mg TeaCrine), and placebo (125mg Maltodextrin). Participants were then assessed for baseline ECG, RHR, BP and PP (energy, feeling of productivity, alertness, desensitization, motivation to do physical tasks, motivation to do mental tasks, and perceived level of focus) using visual analogue scales (VAS; 1-10 scale) every 30 min until 120 min after the first dosage. Following baseline assessments, participants were instructed to consume their supplement upon waking each morning with approximately 12oz of water for four consecutive weeks. VAS measures were repeated after one and two weeks of supplementation, while all baseline assessments were repeated after four weeks of supplementation.

**Results**

Separate 3-way analyses of variance with repeated measures (group x sex x time) revealed no significant interactions. Rather, main effects for time were noted for RHR (p<0.001), BP (systolic: p=0.001; diastolic: p=0.018), and corrected QT interval (p<0.001) where each measure decreased over the 4-week supplementation period, independent of supplementation strategy or sex. Increased (p<0.001) R-R and P-P intervals from pre to 60 min post (across 4-weeks of supplementation), energy, alertness, focus, and motivation to perform physical and mental tasks were also observed on each time point compared to baseline. No adverse events were reported in participants that completed the investigation.

**Conclusion**

These data suggest that Dynamine™ alone or in combination with TeaCrine^®^ does not significantly affect heart rhythm, RHR, BP, and PP following acute or chronic supplementation at the dosages used in this study.

**Acknowledgements**

Compound Solutions, Inc. grant

## A23 The Effect of Time-Restricted Feeding in Combination with Resistance Training on Measures of Body Composition, Muscle Performance, Resting Energy Expenditure, and Blood Biomarkers

### Matthew T. Stratton^1^, Grant M. Tinsley^2^, Michaela Alesi^1^, Garrett M. Hester^1^, Alex A. Olmos^1^, Paul R. Serifini^1^, Andrew Modjeski^1^, Gerald T. Mangine^1^, Kelsey King^1^, Shelby Savage^1^, Austin Webb^1^, and Trisha A. VanDusseldorp^1^

#### ^1^Dept. of Exercise Science and Sport Management, Kennesaw State University, Kennesaw, GA, USA; ^2^Department of Kinesiology & Sport Management, Texas Tech University, Lubbock, TX, USA

##### **Correspondence:** Trisha A. VanDusseldorp (tvanduss@kenensaw.edu)

**Background**

Time-restricted feeding (TRF) limits daily food intake to a 4-10-hour time period. Interest from exercising populations in TRF has grown from recent investigations highlighting decreases in body mass (BM) fat mass (FM) and body fat percentage (BF%) with maintenance of fat-free mass (FFM) and improvements in muscular performance (MP).

**Materials and Methods**

Twenty-six recreationally active males were randomly assigned to one of two groups, a TRF group (n = 13; 22.9 ± 3.6 yrs; 82.0 ± 10.6 kg; 178.1 ± 7.3 cm), consuming all meals within a given 8-hour time period, or normal diet group (ND; n = 13; 22.5 ± 2.2 yrs; 83.3 ± 15.0 kg; 177.5 ± 8.8 cm), who maintained normal meal patterns. Both groups were placed in a 25% calorie deficit and asked to consume 1.8 g/kg/day of dietary protein. Participants underwent 4 weeks of supervised full body resistance training three times per week. Body composition (FM, FFM, BF%) was assessed by 4-compartment model utilizing dual energy X-Ray absorptiometry, air displacement plethysmography, and bioelectrical impedance analysis. Skeletal muscle cross-sectional area (CSA) and muscle thickness (MT) of the vastus lateralis (VL) rectus femoris (RF) and biceps brachii (BB) muscles were examined via ultrasonography. Resting energy expenditure (REE) was assessed via indirect calorimetry. Changes in MP were examined via bench (BP_1RM_) and leg press (LP_1RM_) 1RM, repetitions to failure (BP_RTF_; LP_RTF_) as well as vertical jump height (VJ_HT_) peak power (VJ_PP_) and force (VJ_F)_. Additionally, serum and plasma were collected for the quantification of blood biomarkers: testosterone, cortisol, and adiponectin. Feelings of perceived recovery, daily life stress, energy, desire to eat, hunger, fullness, and motivation to do physical tasks were assessed throughout. A two-way (time × group) repeated measures ANOVA was used to compare groups across time for all variables.

**Results**

Significant decreases (p < 0.05) were noted in BM, FM, BF%, testosterone, adiponectin, and REE, along with significant increases in BP_1RM_, LP_1RM_, VJ_HT_, VJ_PP_, VL_CSA_, BB_CSA_, BB_MT_ in both groups. However, no group × time interactions were noted for any of the preceding variables. Plasma cortisol levels were significantly elevated at post (p = 0.018) only in ND. FFM was maintained equally between groups. No differences were seen for psychometric parameters.

**Conclusion**

Our results suggest that short-term TRF does not enhance reductions in FM as compared to caloric restriction alone. Furthermore, short-term TRF does not hinder improvements in MP or increases in skeletal muscle CSA.

## A24 Quantifying Similarities and Differences Between Wearable Activity Trackers in University Students

### Schumacher, C., Bell, B., Harris, M., Durst, S., Thombs, E., and Lowery, L.

#### Exercise Science Program, University of Mount Union, Alliance, OH, USA

##### **Correspondence:** Lowery, L. (LoweryLM@mountunion.edu)

**Background**

Wearable activity trackers (accelerometers) and caffeinated drinks are both popular among university students. There is thus a need for model-specific information on the agreement of these devices worn by students at different bodily sites and what impact caffeine may have on them. We hypothesized that accelerometers worn at different anatomical sites would agree when assessing daily movements in physically active university students. Further, we hypothesized caffeine intake would correlate positively with the amount of daily movements recorded.

**Materials and Methods**

Fifteen participants wore an Omron HJ-321 pedometer (Omron Healthcare Inc., Lake Forest, IL) at the right hip and a FitBit Charge HR (FitBit Inc., San Francisco, CA) on the right wrist from waking until sleep for four days. During this period, participants completed a diet log that was analyzed for nutrient and caffeine intake using The Food Processor 11.4 (ESHA research, Salem, OR). Statistical analyses were performed using Statistica Software 13.3 (TIBCO Software, Palo Alto, CA).

**Results**

Omron accelerations correlated with those of FitBit (r=0.92; p<0.001), explaining 84.6% of the variance (r^2^=0.846) but systematically underestimated FitBit by 14%. Further, there was a lack of correlation between average dose (mg) of caffeine consumed over four days and average number of accelerations recorded by Omron (r= -0.047, p=0.88) or Fitbit (r= -0.050, p=0.87). Individual-day coffee and caffeine intake similarly did not correlate with specific daily accelerations.

**Conclusions**

Within the limitations of this design, we conclude that waist-worn Omron HJ-321 data correlates highly but systematically underestimates wrist-worn FitBit Charge HR data, and that multiplying Omron HJ-321 data by a factor of 1.14 provides similar information. That is, upper body movements of university students appear to contribute 14% to daily movement. Extrapolations to movement-based energy expenditure should be made with caution, however, because arm movements (made at the wrist) lack the same amount of work as whole-body (waist-recorded) movements and wrist devices may use algorithms to estimate stride length when inferring steps. Secondarily, caffeine intake had no reproducible relationship to daily movements recorded by either device, potentially due to enforced exercise (from athletics coaches) and walking to scheduled classes, which removes an element of (caffeine-influenced) volitional exertion. These may be reasons our data differ from those on free-living middle-aged Australians.(Torquati *et al,* 2018) Future studies on university students should control for team sport exercise by relating coffee/ caffeine intake to movement patterns of narrow athletic cohorts.

**Acknowledgements**

This research was supported in part by the University of Mount Union Inter-Science Research Club and Student Senate Research Fund.

## A25 The Effects of Gender on Psychometric and Epinephrine Responses to Pre-Exercise Coffee

### Harris, M., Putman, R., Ruffner, K., Slack, G., Vansickle, A., Mendel, R., and Lowery, L.

#### Exercise Science Program and Biochemistry Program, University of Mount Union, Alliance, OH, USA

##### **Correspondence:** Lowery, L (LoweryLM@mountunion.edu)

**Background**

Caffeinated drinks are frequently ingested pre-exercise for ergogenic effects. Previous coffee data from our laboratory (Wise, et al. 2014) suggested similar bench press enhancement between sexes. There is, however little research comparing women to men in psychometric and catecholamine responses in this pre-exercise setting. We hypothesized that alertness and epinephrine (EPI) responses to coffee-plus-exercise would not differ between sexes.

**Materials and Methods**

Sixty minutes after randomized ingestion of Starbucks Via instant coffee (VIA) or decaffeinated coffee (DCF), fasted participants (19 men;18 women) engaged in Smith machine bench pressing and squatting at 50% of one-repetition maximum, followed by vertical jumps. Alertness (Likert-type scale 1-5) was assessed and blood draws of a random subset (6 men; 4 women) were taken immediately post-absorption and post-exercise. Samples were analyzed via enzyme-linked immunosorbent assay for EPI. Factorial ANOVA and SNK post-hoc were performed using Statistica Software 13.3 with significance set at ≤0.05 and values of p=0.06-0.10 considered trends.

**Results**

A significant drink-by-sex interaction for alertness occurred across all subjects: Women post-exercise (VIA 4.4±1.3 vs. DCF 3.2±1.7; p≤0.05) vs. men (VIA 4.2±1.8 vs. DCF 3.7±1.7; p<0.05). Covaried for body mass, women still trended toward higher alertness (p<0.10). In the subset with both psychometric and hormonal data, greater alertness enhancement for women was (VIA 4.8±0.5 vs. DCF 3.2±0.5; p<0.05) vs. men (VIA 4.4±0.6 vs. DCF 4.3±1.0; p>0.05). Alertness enhancement was consistent with EPI elevation, which showed a trend (p<0.10): Women VIA 135.9±97.9% vs DCF 5.6±29.7%; men VIA 21.0±87.2% vs. DCF 27.1±95.9%. Covarying for baseline EPI dissolved the trend (p>0.05). A large effect size for EPI percent change was seen for women (2.04) but not men (0.07).

**Conclusion**

The complete data set failed to support our hypothesis that sexes would not differ in alertness after a cup of coffee and exercise. Women became more alert, which persisted as a trend after adjusting for body weight. Further, a subset of women exhibited greater change in alertness which coincided with the statistical trend and large effect size in EPI elevation compared to men. Future research should employ various coffee types, exercise protocols, and serum measurements across all subjects.

**Acknowledgements**

This research was supported in part by the University of Mount Union Inter-Science Research Club and Student Senate Research Fund.

## A26 The Influence of Caloric Expenditure, Heart Rate Responses, and Session-RPE on Muscle Glycogen Content of the Rectus Femoris Muscle Throughout a Division I Volleyball Preseason

### Gabriel J. Sanders^1^, Haley Libs^1^, Brian Boos^1^, Frank Shipley^1^, Corey A. Peacock^2^

#### ^1^Northern Kentucky University, Highland Heights, KY, USA; ^2^Nova Southeastern University, Fort Lauderdale, FL, USA

##### **Correspondence:** Gabriel J. Sanders (sandersg1@nku.edu)

**Background**

Jump loads in volleyball are generally, well understood. However, less is known about the aerobic demands and caloric cost of preseason volleyball training and how it may influence muscle glycogen content. It is reasonable to suggest that excessive preseason training loads may significantly alter muscle contractile properties and even glycogen stores, thus hampering performance. Therefore, the purpose of the study is to assess the influence of caloric expenditure, heart rate responses, and perceived training load on muscle glycogen content measured via high frequency ultrasound throughout a preseason period in division I volleyball athletes.

**Materials and methods**

Thirteen division I female volleyball athletes (18–22 years old) were monitored throughout a preseason training camp that included 13 practices across nine days. Initially, height and weight were measured upon arrival to campus. Then each morning, while seated in the supine position with both legs straight, athletes’ right and left rectus femoris (RF) were scanned using high frequency ultrasound (Phillips Lumify L12-4 Transducer, Bothell, WA, USA). The scanned images were analyzed with validated MuscleSound® software (MuscleSound, Glendale, CO, USA) to calculate a muscle fuel rating score (glycogen content) by detecting variations in the greyscale ultrasound image of the RF muscle. The software isolates the muscle fibers of the RF and calculates the mean pixel intensity (greyscale brightness) of the scans to create a muscle fuel rating arbitrary unit (AU) ranging from 0–100. Then, throughout each practice, athletes wore chest electrode heart rate devices (Polar Team Pro System, Polar Global, Kempele, Finland) to record caloric expenditure, average heart rate (HR_avg_), and maximum heart rate (HR_max_). To enhance the accuracy of caloric expenditure estimations, age, height, weight, and HR_max_ from the conditioning test were entered in to the Polar software. Lastly, at the end of each practice, athletes reported their RPE and RPE was then multiplied by the practice duration to produce a training load metric called session-RPE (s-RPE).

**Results.** A forward regression analyzed if calories (1699 ± 981 kcals), HR_avg_ (132 ± 14 BPM), HR_max_ (184 ± 11 BPM), and s-RPE (1689 ± 942) accounted for variations in muscle fuel ratings (42 ± 16 AU) throughout the preseason. Muscle fuel rating was significantly, negatively related to caloric expenditure in practice (*r* = -.405, *p* < 0.001). Caloric expenditure was a significant predictor of glycogen content (*β* = - 0.007, *p* < 0.001), however, HR_avg_ (*β* = -.192, *p* = 0.102), HR_max_ (*β* = -.080, *p* = 0.388), and s-RPE (*β* = .157, *p* = 0.273) were not significant predictors.

**Conclusions**

Caloric expenditure is a significant predictor when monitoring changes in muscle fuel ratings (glycogen content) throughout a volleyball preseason. High frequency ultrasound is a non-invasive tool that can measure glycogen content and may help practitioners objectively monitor athlete nutrition and even hydration.

## A27 The effects of a multi-ingredient pre-workout supplement on cycle ergometry performance during a 3-minute all-out test

### Taylor K. Dinyer^1^, M. Travis Byrd^1^, Pasquale J. Succi^1^, Brian J. Wallace^2^, Haley C. Bergstrom^1^

#### ^1^Department of Kinesiology and Health Promotion, University of Kentucky, Lexington, KY, USA; ^2^Department of Kinesiology, University of Wisconsin Oshkosh, Oshkosh, WI, USA

##### **Correspondence:** Taylor K. Dinyer (taylor.dinyer@uky.edu)

**Background**

The 3-min all-out test provides estimates of aerobic (critical power [CP]) and anaerobic (anaerobic work capacity [AWC]; peak power [PP]) energy system capacities, and average power output (mean power [MP]). The purpose of this study was to examine the effects of a multi-ingredient pre-workout supplement on aerobic and anaerobic parameters during a 3-min all-out test.

**Materials and Methods**

Thirteen men (Age: 22±2 yrs; Height: 177.8±8.0 cm; Weight: 84.5±12.9 kg) participated in this double-blind, placebo-controlled study. The subjects performed 2 separate 3-min all-out tests on the cycle ergometer at a resistance set at 4.5% of body weight. Thirty minutes prior to the test, the subjects ingested either a placebo (PL) or supplement (SUP) containing citrulline malate (6g), leucine (4g), aspartic acid (3g), creatine hydrochloride (2g), beta-alanine (1.6g), tyrosine (1.2g), and caffeine anhydrous (350mg). The CP was the average power output over the final 30-sec of the test (150-180 sec) and the AWC reflected the work done above CP using the following equation: AWC = 150 sec (P_150_ – CP), where P_150_ is the average power output for the first 150 sec of the test. Mean power and PP were calculated as the average power output over 180-sec and the highest 5-sec power output reached during the test, respectively. Separate, paired samples t-tests were used to compare CP, AWC, PP, and MP between the PL and SUP trials at an alpha level of p≤0.05.

**Results**

Paired samples t-tests indicated no difference between the PL and SUP trials for CP (PL: 212±32 W; SUP: 216±33 W; p=0.277), AWC (PL: 15.0±2.9 kJ; SUP: 15.6±3.1 kJ; p=0.258), and PP (PL: 750±136 W; SUP: 751±134 W; p=0.943). However, the MP was greater (p=0.037) for the SUP (302±40 W) compared to the PL (295±35 W) trial.

**Conclusions**

The multi-ingredient pre-workout supplement examined in this study did not affect parameters (CP, AWC, or PP) reflective of specific energy systems, but did result in an increase in MP, compared to the PL. These effects may be related to caffeine’s role as an adenosine receptor antagonist and decreases in the perception of effort over 3-min that were not reflected in the aerobic and anaerobic parameters (CP, AWC, and PP) defined from shorter epochs during the test. Thus, ingestion of a pre-workout supplement prior to high-intensity activities of at least 3 min may decrease perception of effort that allows for maintenance of a higher average power output.

**Acknowledgements**

The supplement examined in this study was provided by MusclePharm Inc. (MusclePharm, Denver, CO) and the placebo was provided by True Nutrition (True Nutrition, Vista, CA).

## A28 Strength and self-perceived body image improvement after l-leucine plus resistance training intervention in mid-life women

### LesLee Funderburk^1^, Jay Yoo^1^, Darryn Willoughby^2^

#### ^1^Family & Consumer Sciences, Robbins College of Health & Human Sciences, Baylor University, Waco, TX, USA; ^2^Health, Human Performance & Recreation, Robbins College of Health & Human Sciences, Baylor University, Waco, TX, USA

##### **Correspondence:** LesLee Funderburk (leslee_funderburk@baylor.edu)

**Background**

This study investigated the effects of l-leucine (leu) supplementation with resistance training (RT) in untrained mid-life women on strength, lean body mass, and body image.

**Materials and Methods**

This was a randomized, double-blind, placebo-controlled trial, in which thirty-five untrained women were randomly assigned to either a 5 g leu or 5 g placebo supplement group coupled with 10 weeks of RT. Subjects were instructed to follow their normal dietary intake. Self-recorded food records were analyzed at baseline and end of study and compared to their total daily energy expenditure (TDEE) calculated via an estimation equation, to determine whether participants were hypo- or hyper-caloric.

*Measures of outcome:* Strength, lean body mass, and body image were assessed before and after intervention. Data were analyzed by utilizing separate 2 x 2 [Group x Time (Pre-Test and Post-Test)] factorial analyses of variance with repeated measures and t-tests (*p* ≤ .05).

**Results**

There were significant increases in strength in both groups in response to RT (p < 0.05), but not supplementation, with no significant increases or differences between groups in lean body mass in response to supplementation. Body image scores significantly improved from baseline in both groups (p < 0.05), with no significant differences between groups. Total kcals at baseline and end of study ranged from 1471 – 1642/day and as compared to TDEE, indicated that subjects were hypocaloric at the time the dietary analyses were completed.

**Conclusion**

Mid-life women had significant increases in strength due to RT. This is an important finding, as they were estimated to be hypocaloric throughout study. Self-perceived body image improved by end of study, presumably associated with the increases in strength versus supplementation.

## A29 Acute testosterone administration does not affect muscle anabolism

### David D. Church^1^, Stefan M. Pasiakos^2^, Robert R. Wolfe^1^, and Arny A. Ferrando^1^

#### ^1^Department of Geriatrics, Donald W. Reynolds Institute on Aging, Center for Translational Research in Aging & Longevity, University of Arkansas for Medical Sciences, Little Rock, AR, USA; ^2^Military Nutrition Division, U.S. Army Research Institute of Environmental Medicine, Natick, MA, USA

##### **Correspondence:** David D. Church (dchurch@uams.edu)

**Background**

The effects of exogenous testosterone (T) administration on muscle protein anabolism and lean body mass accrual are well established. The muscle protein kinetic mechanisms through which T administration improves anabolism are less appreciated. Fasted net muscle protein balance is improved in healthy males following a 5d treatment with an oral T analogue or T injection. In the post-absorptive state, the essential amino acid (AA) precursors for protein synthesis (PS) at the whole body level are derived entirely from protein breakdown (PB). In certain tissues and organs, the precursors for PS can be derived from uptake of circulating AA. Improved synthetic efficiency of muscle protein in the post-absorptive state in response to T refers to an increase in the rate of PS relative to the rates of PB and inward AA transport. Whether the effects of exogenous T on muscle protein kinetics occur acutely upon exposure is not known.

**Materials and Methods**

We investigated the effects of acute T exposure on leg muscle protein kinetics and selected AA transport using the arteriovenous balance model, and direct calculations of mixed-muscle protein fractional synthesis (FSR) and breakdown (FBR) rates. Four healthy men were studied over a 5 hour period with and without T; infusion rate= 0.25 mg·min^-1^, providing 30mg of T over the course of the study. Free testosterone concentrations in serum were determined by a double antibody method with commercial radio immunoassays (Diagnostic Products, Los Angeles, CA), the intra-assay CV was 2.9%. The area under the curve (AUC) throughout the entire infusion protocol (0 to 5 hr) was calculated using the trapezoidal method. Data are presented as means ± SEM. All variables were compared by paired samples t-test with statistical significance designated at α ≤ 0.05.

**Results**

Muscle protein FSR, FBR, and net protein balance (direct measures and model derived) were not affected by T, despite a significant increases in arterial (p = 0.009) and venous (p = 0.064) free T area under the curve during T infusion. T infusion had minimal effects on AA transport kinetics.

**Conclusions**

These data indicate that exposing skeletal muscle to T does not confer immediate effects on AA kinetics or muscle anabolism. There remains an uncertainty as to the earliest discernable effects of T on skeletal muscle protein kinetics after initial administration.

## A30 The effects of specific collagen peptides on sports-related activities in patients with chronic ankle instability

### Denise Zdzieblik^1,2^, Patrick Jendricke^1^, Steffen Oesser^2^, Albert. Gollhofer^1^, Daniel König^1^,

#### ^1^Department of Sport and Sport Science, University of Freiburg, Freiburg, Germany; ^2^CRI, Collagen Research Institute GmbH Kiel, Germany

##### **Correspondence:** Denise Zdzieblik (denise.zdzieblik@cri-mail.org)

**Background**

Chronic ankle instability (CAI) is quite common among competitive athletes, and there is still a need for better therapeutic approaches to prevent or treat the condition. In a previous investigation, specific collagen peptides (TENDOFORTE®) were shown to enhance the perceived function of the ankle as measured by the Cumberland Ankle Instability Tool (CAIT), and the Foot and Ankle Ability Measure (FAAM; German Version) [1].

The objective of this study was to perform a more detailed exploratory examination of the data from the study by Dressler et al. [1] to gain a greater understanding of the impact of TENDOFORTE® on CAI patients.

**Materials and Methods**

The statistical analysis consisted of data from 50 male and female athletes with CAI (26.9 ± 9.1 years). Over a period of 6 months, the participants received either 5 g TENDOFORTE® or 5 g placebo (maltodextrin). In addition, all the subjects were asked to follow a standardized ankle-loading protocol. FAAM-G and CAIT questionnaires were obtained at baseline (T_0_) and 6 months (T_6_).

**Results**

Overall, the CAIT and FAAM scores improved in the group receiving collagen peptides to a statistically significantly greater extent than in the placebo group (p < 0.05). When considering each item, the improvements were reported mainly for sporting activities by the FAAM subscale “Sports” (Figure). With the exception of “Performing with proper techniques” (p = 0.060), the improvements for all the items were statistically significantly greater in the treatment group than the placebo group (p < 0.05). Furthermore, CAIT scores in the treatment group improved in all items by 9 – 33 %. In contrast, only 7 of 9 CAIT items improved in the placebo group by 2 – 22 %.

**Conclusion**

These findings demonstrate that the daily intake of 5 g specific collagen peptides (TENDOFORTE®) was associated significantly with improvements in sports-related activities among patients suffering from chronic ankle instability.

**Fig. 1 (abstract A30).**
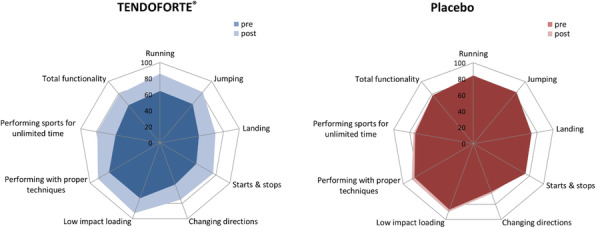
Percentage changes in the FAAM subscale scores “Sports” (n = 50)

**Acknowledgement**

The authors have neither financial nor competing interests concerning the outcome of this investigation.

**Reference:**

1. Dressler, P., Gehring, D., Zdzieblik, D., Oesser, S., Gollhofer, A., and König, D. **Improvement of Functional Ankle Properties Following Supplementation with Specific Collagen Peptides in Athletes with Chronic Ankle Instability**. *J. Sports Sci. Med.* 2018 **17**, 298–304.

## A31 Improved Muscle Protein Synthesis is Achieved with 3.6g of Free Form Essential Amino Acid Ingestion in Elderly

### David D. Church, Arny A. Ferrando, and Robert R. Wolfe

#### ^1^Department of Geriatrics, Donald W. Reynolds Institute on Aging, Center for Translational Research in Aging & Longevity, University of Arkansas for Medical Sciences, Little Rock, AR, USA

##### **Correspondence:** David D. Church (dchurch@uams.edu)

**Background**

It is well-established that the essential amino acid (EAA) component of protein is responsible for the stimulation of muscle protein synthesis. Ingestion of free-form EAA produce a robust increase in muscle protein synthesis. In younger subjects, studies have shown that a dose as small as 3g of EAA can stimulate muscle protein synthesis, with a maximal response elicited by ingesting 15g. However, there is a reported anabolic resistance to protein in older populations. Thus, we sought to determine if submaximal doses of free-form EAA would stimulate muscle protein synthesis in older volunteers.

**Materials and Methods**

Eleven subjects (67 ± 6 (SD) years; 5M: 6F) received 3.6g and 12 subjects (68 ± 5 years; 8M: 4F) received 10.8g of an EAA formula. Subjects were studied for 2 hours in the fasted state and 3 hours after EAA ingestion. Muscle biopsies were taken at 3, 5, and 8 hours after initiation of tracer infusion to determine fractional rate of protein synthesis (FSR). Blood draw were obtained between hours 5 and 8 to calculate the area under the curve (AUC) response for total EAA’s. Data are presented as means ± SD. A group × time repeated measures analysis of variance was used to analyze muscle FSR changes. Independent samples t-test were used to analyze AUC measures with statistical significance designated at α ≤ 0.05.

**Results**

A significant main effect of time (p ≤ 0.001) was observed for muscle FSR with both the 3.6g (0.0568 ± 0.0338%/hr) and the 10.8g (0.0691 ± 0.0412%/hr) increasing from the fasted period to the fed period. However, the increase in the muscle FSR response was not significantly different between the two conditions (p = 0.921). The EAA AUC was significantly greater following the 10.8g (272.7 mol/L/min) as compared to the 3.6g (185.4 mol/L/min).

**Conclusions**

Despite a significantly higher EAA AUC the muscle FSR response was not significantly enhanced by 10.8g as compared to 3.6g of free-form EAA’s. Therefore, ingestion of 3.8g of free-form EAA’s appears to be a viable way to maximize protein synthesis in elderly subjects.

## A32 The effects of caffeine gum on heart rate variability before and after exercise

### Emily J Ryan^1^, Tareq, Shahbal^2^, Kayla Klett^2^, Jason Edsall^3^, Andres E. Carrillo^3^ and Edward J. Ryan^3^

#### ^1^West Virginia University, Morgantown, WV, USA; ^2^Department of Biology, Chatham University, Pittsburgh, PA, USA; ^3^Department of Movement Science, Chatham University, Pittsburgh, PA, USA

##### **Correspondence:** Emily J Ryan (ejfickes@hsc.wvu.edu)

**Background**

Low Heart Rate Variability is resultant from increased Sympathetic input and/or decreased parasympathetic input and has been associated with an increase in risk of cardiac mortality Caffeine is widely used by recreational athletes to enhance performance. The purpose of the present study was to determine the effects of a lower dose of caffeine on heart rate variability before and after exercise in recreationally active adults.

**Materials and methods**

Nine apparently healthy, younger (23 ± 4 years) adults participated in three separate laboratory sessions. During the first visit, subjects underwent a submaximal single staged treadmill test to predict maximal oxygen consumption (VO_2max_). For the next two visits (experimental testing) subjects arrived at the Exercise Science Laboratory between 0600 and 1200 hours, were fitted with a Polar V800 heart rate monitor, and sat quietly for 10 min for baseline assessment of heart rate variability. Thereafter two pieces of Military Energy Gum [caffeine (CAFF) or placebo (PLA)] were administered in a double-blind manner. Subjects were instructed to chew the gum for 5 min then expectorate. Following a standard warm-up, subjects walked at 60% VO_2max_ for 20 min. Subjects then immediately sat and rested for 30 min for heart rate variability assessment. R-R interval data were downloaded to a PC and heart rate variability indices were assessed via Kubios HRV Standard software.

**Results**

Heart rate variability indices data were analyzed via a 2 (treatment) by 4 (time) analysis of variance(ANOVA). The ANOVA demonstrated a main effect of treatment for an increase in the Root Mean Square of Successive Differences (RMSSD) (p = .004). No main effect of treatment or treatment by time interaction was noted for all other indices assessed (p ≥ 0.110).

**Conclusions**

These data demonstrated that a modest dose of caffeine improved RMSSD before and after exercise in recreational adults.

## A33 Effects of Curcumin and Piperine on Delayed Onset Muscle Soreness and Perceived Exertion of a Muscle Damaging Protocol

### Thomas D. Cardaci^1^, Steven B. Machek^1^, Paul S. Hwang^1^, Dylan T. Wilburn^1^, Emiliya S. Suezaki^1^, Darryn S. Willoughby^1^, FISSN

#### ^1^Exercise & Biochemical Nutrition Laboratory, Department of Health, Human Performance, & Recreation, Robbins College of Health and Human Sciences, Baylor University, Waco, TX, USA

##### **Correspondence:** Darryn S. Willoughby (darryn_willoughby@baylor.edu)

**Background**

Curcumin is a natural polyphenolic compound extracted from turmeric commonly combined with piperine (to increase bioavailability). Due to its profound antioxidant and anti-inflammatory properties, curcumin supplementation has become popular amongst athletes to treat skeletal muscle inflammation and delayed onset muscle soreness (DOMS). However, data to show curcumin’s ability to attenuate DOMS is inconclusive. Therefore, the purpose of this study was to investigate the effects of curcumin and piperine on DOMS following exercise induced muscle damage. A secondary purpose of this study was to investigate curcumin and piperine’s effect on perceived exertion (RPE) of the exercise bout.

**Materials and Methods**

Twenty-three recreationally active male and female participants were randomized into two groups (Curcumin + Piperine [n=11]; or Placebo + Piperine [n=12]). Both groups were instructed to consume 2g of their respective supplement and 20mg of piperine for eleven consecutive days. Following eight consecutive days of supplementation, participants performed a 45-min eccentrically biased muscle damaging treadmill protocol at 60% VO_2max_. Using the Borg Scale (6-20), RPE was monitored in 5 min intervals during the muscle damage protocol. Participants reported DOMS by drawing an intersecting mark across a continuum line extending from 0 to 13 (0 = no soreness, 13 = extreme soreness) immediately before and 3-, 24-, 48-, and 72-hours post muscle damage protocol. A 2x5 mixed model ANOVA with pairwise comparisons (DOMS) and an independent T-test (RPE) were conducted with significance set at P<0.05.

**Results**

No significant difference in average RPE (*t*_(21)_=0.26, p = .979) was found between placebo + piperine 14.72 (±1.57) vs. 14.74 (±2.16) curcumin + piperine conditions. No significant differences in DOMS was observed at any time points between conditions (*F*_(1, 20)_= 0.42, p = .524, η^2^ = .021). However, there was a significant time effect for DOMS (*F*_(4, 20)_= 29.6, p < .001, η^2^ = .597). Pairwise comparisons indicated that DOMS was significantly greater from baseline at all time points except 72 hours post exercise induced muscle damage (p = .082).

**Conclusions**

The muscle damage protocol appears to induce DOMS 3-, 24-, and 48-hours post exercise with no differences between groups. Therefore, curcumin and piperine did not mitigate participant perceived muscle soreness. Moreover, equivocal RPE between groups indicate no differences in participant perceived exertion of the muscle damage protocol. While these subjective indicators provide insight, more objective/measurable markers may establish a more comprehensive mechanism of curcumin and piperine’s effects on skeletal muscle damage.

## A34 Effects of Carbohydrate Supplementation on Resistance Exercise Performance and Index of Fatigue

### Dylan T. Wilburn^1^, Thomas D. Cardaci^1^, Steven B. Machek^1^, Paul S. Hwang^1^, Darryn S. Willoughby^1^, FISSN

#### ^1^Exercise & Biochemical Nutrition Laboratory, Department of Health, Human Performance, & Recreation, Robbins College of Health and Human Sciences, Baylor University, Waco, TX, USA

##### **Correspondence:** Darryn S. Willoughby (darryn_willoughby@baylor.edu)

**Background**

The positive effects of carbohydrate supplementation and aerobic exercise has been studied extensively over the past century. There have been few studies that view changes in performance and recovery responses to carbohydrate supplementation with exhaustive resistance exercise. Therefore, the purpose of this study was to investigate how carbohydrate influences resistance exercise performance.

**Materials and Methods**

Ten apparently healthy male individuals with at least a year of resistance exercise experience were recruited for this study. The mean (±SD) age, mass, and body fat percentage was 21 (±2.27) years, 90.4 (±19.3) kg, and 20 (±8.5) %, respectively. The first visit consisted of baseline preliminary test to determine 1-RM on angled leg press, a 30 min recovery, followed by four sets of reps to fatigue protocol at 70% of that 1-RM. The reps to fatigue protocol consisted of four sets to failure separated by 45 s of rest. Foot positioning and knee angle ROM were standardized for each participant for each visit. For visits 2 and 3, participants were divided in a randomized, double-blind, cross-over experimental design, participants completed the same reps to fatigue bout after ingesting either placebo or carbohydrate. At the start of each visit, participants ingested either a maltodextrin carbohydrate supplement at 2 g/kg body mass or non-caloric, identically-tasting placebo, and waited 30 min before completing the four sets of reps to fatigue. A 2x4 repeated measures ANOVA and a pair samples t-test were conducted at a significance level of p < .05.

**Results**

No significant difference in total reps to fatigue (*t*_(9)_=-1.47, p = .174) was found between placebo 53.8 (±7.8) vs. 51.8 (±6.9) carbohydrate conditions. No significant difference in reps completed was seen between conditions within each individual set (*F*_(1, 9)_= 2.169, p = .175, η^2^ = .194). However, there was a significant difference in reps completed between sets for both conditions (*F*_(3, 9)_= 126.1, p < .001, η^2^ = .933). Pairwise comparisons indicated that there was a significant decrease in reps completed between all sequential sets in both conditions, except sets 3 and 4 (p = .212).

**Conclusions**

Carbohydrate did not appear to have any effect on resistance exercise performance when completed to fatigue. Similarly, carbohydrate consumption did not alter recovery between sets which is indicated by the total reps to fatigue within each set being identical between conditions. This indicates that carbohydrate did not have any impact on performance or acute inter-set recovery during the exhaustive resistance exercise bout.

## A35 Fat-Free Mass and Knee Extensor Peak Torque At Pre Are Predictive of Changes in Squat Max Following 10 Weeks of Moderate-to-low-volume, High-load Resistance Training in College Aged Men

### Casey L. Sexton, Christopher G. Vann, Shelby C. Osburn, Paul A. Roberson, Carlton D. Fox, McLelland-Rae Johnson, Jacob Z. Shake, Michael D. Roberts

#### School of Kinesiology, Auburn University, Auburn, AL, USA

##### **Correspondence:** Michael D. Roberts (mdr0024@auburn.edu)

**Background**

Different paradigms of load and volume are selected during training so that a specific fitness characteristic can be improved, and high-load training has generally been characterized by the frequent use of loads exceeding 85% of a 1RM with the aim of increasing strength. When loads of this magnitude are used, lower volume is often implemented due to acute fatigue to the PCr pathway, because of which we would not expect to see concomitant increases in fat-free mass. The purpose of this study was to determine the effects of 10 weeks of high-load, moderate- to low-volume training on changes in fat-free mass and estimated 1RM back squat. Additionally we sought to investigate if PRE knee extensor peak torque at 60 and 120 deg/sec and PRE fat-free mass were predictive of back squat performance following this intervention.

**Materials and Methods**

16 recreationally trained, college-aged males completed a 10 week self-reported, high-load, moderate- to low- volume training program. At PRE and POST training, participants performed a series of tests including bio-electrical impedance spectroscopy (BIS) to determine fat-free mass, isokinetic dynamometry at 60 and 120 deg/sec to determine knee extension peak torque, and 3RM back squat to estimate maximal lower body strength.

**Results**

PRE fat-free mass trended to be different from POST fat-free mass (p = 0.054). PRE knee extension peak torque at 60 deg/sec explained ~31% of the variance in POST estimated 1RM back squat ( r 2 = 0.312, p = 0.025). PRE knee extensor peak torque at 120 deg/sec explained ~33% of the variance in POST estimated 1RM back squat (r 2 = 0.329, p = 0.020). PRE fat-free mass explained ~37% of the variance in POST estimated 1RM back squat ( r 2 = 0.375, p = 0.012).

**Conclusions**

Fat-free mass does not seem to change with high-load, moderate- to low-volume training. PRE training knee extension peak torque at 60 and 120 deg/sec and PRE fat-free mass are possibly predictive of POST training estimated 1RM back squat in this population.

## A36 Total Creatine and Creatine-Associated Markers in Relation to Wilks Coefficient in Both Male and Female Powerlifters

### Steven B. Machek, Paul S. Hwang, Thomas D. Cardaci, Emiliya S. Suezaki, Caelin Kim, Dylan T. Wilburn, & Darryn S. Willoughby, FISSN

#### ^1^ Exercise & Biochemical Nutrition Laboratory, Department of Health, Human Performance, & Recreation, Robbins College of Health and Human Sciences, Baylor University, Waco, TX, USA

##### **Correspondence:** Darryn S. Willoughby (darryn_willoughby@baylor.edu)

**Background**

Powerlifting is a barbell sport consisting of the squat, bench press, and deadlift. Powerlifting training and competition is highly dependent on the immediate energy pathway, where adenosine triphosphate (ATP) is made available by the ATP-phosphocreatine system. In Powerlifting competition, the Wilks Coefficient is a popular and common means to normalize performance relative to bodyweight. Therefore, the purpose of this study was *1)* to compare various markers associated with creatine content to Powerlifter Wilks coefficient, and *2)* compare these markers between genders.

**Materials and Methods**

Twelve actively competing Powerlifters (PL; n = 6M/6F) and twelve sedentary controls (CON; n = 6M/6F) were recruited for this cross-sectional analysis. After providing consent and dietary logs, subjects donated ~20mL blood and underwent a percutaneous muscle biopsy from the *vastus lateralis* using the fine needle aspiration method. Dietary macronutrients, serum total creatine (STC), relative serum creatinine (rCRT), muscle total creatine (MTC), and muscle creatine transporter (SLC6A8) were analyzed via multiple 2x2 (group x gender) analysis of variance (ANOVA) at a significance of p<0.05. MTC was compared to Wilks Coefficient using Pearson correlation coefficients at a significance of p<0.05. In the case of trending significance (p<0.10), PL was further divided into intermediate (PLI; n=5) and advanced (PLA; n=7) groups based on mean Wilks Coefficient. A 3x2 (group [PLA, PLI, CON] x gender) and a 2x2 (group [PLA, PLI] x gender) ANOVA were employed to detect differences between all groups and between Powerlifters, respectively.

**Results**

There were no significant group, gender, or interaction effects between PL and CON for relative STC, MTC, or SLC6A8. Serum rCRT revealed a significant gender effect across all three groups (p=0.009), as well as a significant group (p<0.001), gender (p=0.004), and interaction effect (p=0.013) between PLI and PLA. Females overall demonstrated greater rCRT verus all groups, and female PLI had higher rCRT versus PLA of either gender. Pearson correlation analysis revealed non-significant, low-moderate correlation between Wilks coefficient and MTC (r = 0.488; p = 0.108).

**Conclusions**

These results imply markers of creatine in both muscle and serum are not predictive of performance in Powerlifting, defined by Wilks coefficient. Overall, variations in Powerlifting performance across gender and skill do not convincingly result from changes in biochemical differences related to creatine metabolism. Therefore, these findings suggest other biochemical markers (fiber type, enzymatic differences, etc.) and/or neurological efficiency underline variations in Powerlifting skill.

**Acknowledgments**

This poster uses data from the OpenPowerlifting project, https://www.openpowerlifting.org. You may download a copy of the data at http://gitlab.com/openpowerlifting/opl-data.

## A37 Applications and perspectives of the RPE clamp protocol during resistance training to investigate ergogenic aids

### Joshua L. Keller, Terry J. Housh, Ethan C. Hill, Cory M. Smith, Richard J. Schmidt, Glen O. Johnson

#### University of Nebraska – Lincoln, Department of Nutrition and Health Sciences, Human Performance Laboratory, Lincoln, NE, USA

##### **Correspondence:** Joshua L. Keller (joshua.keller@huskers.unl.edu)

**Background**

Sports nutrition-related investigations have previously examined the ability to mitigate performance fatigability during various exercises. In addition, previous investigations have utilized the RPE clamp protocol to examine the efficacy of ergogenic aids during aerobic exercise, but not resistance training. Therefore, the purpose of this study was to examine potential applications of the RPE clamp model and sex-related differences in performance fatigability as a result of a sustained isometric leg extension muscle action anchored to RPE=5.

**Materials and Methods**

Twenty college-aged adults (10 men: 22.9 ± 2.0 yrs, 180.6 ± 7.3 cm, 79.8 ± 8.5 kg and 10 women: 23.1 ± 2.3 yrs, 172.5 ± 10.1 cm, 77.1 ± 26.6 kg) performed sustained muscle actions at RPE=5 for a maximal duration of 5-min. The MVICs were performed prior to (pretest) and following (posttest) the sustained isometric muscle actions. The force values were calculated every 5% of the actual time-limit (20 total timepoints) and normalized to pretest MVIC. A 2 (Sex: Men, Women) × 2 (Test: Pretest, Posttest) mixed factorial ANOVA was used to examine mean differences in performance fatigability. Regression and slope analyses were used to examine the patterns of force decline for the men and women.

**Results**

There was no significant (p=0.28) interaction, but there were significant main effects for Test (p<0.001; ${\upeta}_{\mathrm{p}}^2$=0.735) and Sex (p=0.008; ${\upeta}_{\mathrm{p}}^2$=0.329). The follow-up t-tests for decreases in force pretest to posttest (performance fatigability) were significant for the men (62.9 ± 14.4 kg to 48.3 ± 12.9 kg; p<0.001, d=1.07) and women (47.3 ± 8.9 to 36.7 ± 5.3 kg; p=0.003, d=1.45). In addition, the pretest MVIC was significantly (p=0.01; d=1.30) greater for the men than women. During the sustained muscle action, there was a significantly (p=0.02) greater linear rate of force decline for the men (b ± SE: -0.13 ± 0.01; r^2^=0.88) than the women (-0.10 ± 0.01; r^2^=0.90).

**Conclusions**

In conclusion, the men were stronger than the women and demonstrated a significantly greater magnitude as well as rate of performance fatigability than the women during the sustained muscle action anchored to RPE=5. Thus, the RPE clamp provides a feasible methodology to examine the efficacy of various ergogenic aids as well as their potential associated sex-specific responses. Future studies should aim to reduce the magnitude and/or rate of performance fatigability with the implementation of an ergogenic aid.

**Acknowledgements**

We would like to thank all the subjects for their participation.

## A38 The rate of fatigue during unilateral versus bilateral, maximal, isokinetic leg extensions

### John Paul Anders^1^, Joshua Keller^1^, Cory Smith^2^, Ethan Hill^3^, Terry Housh^1^, Glen Johnson^1^, Richard Schmidt^1^

#### ^1^Department of Human Sciences, University of Nebraska- Lincoln, Lincoln, NE, USA ; ^2^College of Health Sciences, Kinesiology, University of Texas at El Paso, El Paso, TX, USA; ^3^School of Kinesiology & Physical Therapy, Division of Kinesiology, University of Central Florida, Orlando, FL, USA

##### **Correspondence:** John Paul Anders (janders@huskers.unl.edu)

**Background**

Fatigue is the inability to maintain performance over time. For athletes, success is largely predicated on their ability to mitigate fatigue and optimize performance throughout a task. Many sport supplements are designed to improve athletic performance by delaying the process of fatigue under various exercise conditions. The purpose of this study was to examine the characteristics of fatigue during maximal unilateral and bilateral isokinetic leg extensions.

**Materials and Methods**

Ten men (X̅ ± SD; age 22.9 ± 3.7 yrs; weight 80.4 ± 7.9 kg; height 177.8 ± 6.7 cm) volunteered to participate in this study. After a familiarization visit, the subjects visited the laboratory on 3 different occasions, separated by a least 48 hours. After a warm up of 5 submaximal isokinetic leg extensions, the subjects performed 2 maximal isokinetic leg extensions at 180 ° s^-1^ to determine peak torque (PT) of the right leg only (RO), left leg only (LO), and bilaterally (BL), in randomized order. The assessment of PT was followed by 50 consecutive, fatiguing, maximal leg extensions at 180 ° s^-1^. Each test visit randomized the fatiguing task to be performed for the RO, LO, or BL condition. Each repetition was normalized to the highest pre-fatigued PT value. Linear regression and comparisons of the slope coefficients were conducted to determine if there were significant differences in the rates of fatigue between the unilateral and bilateral conditions.

**Results**

There were significant linear decreases (*p*< 0.001) in normalized torque for RO (r^2^ = 0.94), LO (r^2^ = 0.96) and BL (r^2^ = 0.90). The rate of fatigue was significantly greater (*p* < 0.001) for the RO (*b*= -1.04 ± 0.04) and LO (*b*= -1.10 ± 0.03) than the BL condition (*b* = -0.54 ± 0.03). The rate of fatigue was also significantly greater (*p* = 0.017) for the LO than the RO condition.

**Conclusions**

The results of this study demonstrated that 50 maximal, isokinetic leg extensions at 180 ° s^-1^ resulted in 50 (RO) to 52% (LO) greater rates of fatigue when performed unilaterally compared to bilaterally. Future research can use these methods to determine the ergogenic efficacy of supplements aimed at reducing the rate of fatigue. Furthermore, these investigations can be tailored to mode-specific conditions (i.e. unilateral or bilateral) best suited to represent an athlete’s needs.

## A39 Effects of carbohydrates and soluble milk proteins supplements on physical performance and muscle damages during a simulated rugby seven tournament: a double-blind, randomized, placebo-controlled, cross-over study

### Mathilde Guerville^1^, Marina Fabre^2,3^, Bertrand Mathieu^4^, Cédric Leduc^4^, Eve Tiollier^2^, Matthieu Clauss^2^, Alexandre Marchand^5^, Julien Robineau^4^, Tanguy Serenari^2^, Julien Piscione^4^, Victoire Visseaux^6^, Jacqueline Brasy^1^, Pascale Le Ruyet^1^, Xavier Bigard^2,7^

#### ^1^Research and Development Lactalis, Retiers, France; ^2^Unité Recherche Laboratoire Sport, Expertise et Performance (SEP), INSEP, Paris, France; ^3^Performance Department, Paris Saint-Germain Football Club, Saint-Germain-en-Laye, France; ^4^French Rugby Federation, Marcoussis, France ; ^5^Agence Française de Lutte contre le Dopage, Paris, France ; ^6^Lactalis Ingredients USA, Buffalo, NY, USA; ^7^Union Cycliste Internationale, Aigle, Switzerland

##### **Correspondence:** Eve Tiollier (eve.tiollier@insep.fr)

**Background**

The objective of this study was to determine the effects of a recovery drink comprising soluble milk proteins and carbohydrates for maintaining physical performance during a simulated rugby seven tournament.

**Materials and Methods**

12 well-trained male rugby players achieved a typical day of a rugby seven tournament, with the achievement of the sequence of 3 simulated matches, intersected with 2 hours of recovery [1]. They performed the same protocol for 3 consecutive weekends. The only difference between each day of rugby tournament was the beverage consumed- in a cross-over double-blind manner- after each simulated rugby match (two 7-min halves). The drinks tested were either a placebo drink (PLA, water), a carbohydrate drink (CHO, 80g of carbohydrates) or an iso-energetic carbohydrates-proteins drink (P-CHO, 20g of pronativ® and 60g of carbohydrates). Physical performance (speed, endurance and power) were measured before the first match (PRE) and after the third match of the day (POST). Muscular-damages markers (specific muscular targeted microRNAs, plasma Creatine-Phosphokinase (CPK), and muscle pain assessment by VAS) were measured before and after simulated tournament at different time points: PRE, POST, after 6 hours and 12 hours of recovery (POST6 and POST12). In order to assess the practical meaning of the results, data were analyzed using the magnitude-based inference approach [2].

**Results**

Regarding physical performance at the end of the day (PRE-POST), the P-CHO condition appeared to have a *likely moderate to large* positive effect on the running velocity from 30m to 50m, compared to the PLA condition. The CHO condition shows similar effects although of lesser magnitude (*likely small to moderate* positive effect). At POST, CHO and P-CHO beverages *probably* improve endurance capacity compared to PLA (*small*).Regarding muscular damages markers, the P-CHO beverage *probably* attenuates the CPK increase observed at POST6 compared to PLA (*moderate*). Both P-CHO and CHO *very likely* and *likely*, respectively, lowered the increase of miR-1 (marker of muscle damage) at POST compared to PLA (*moderate)*. Finally, P-CHO drinks reduces muscular pain at palpation and pain during stairs descending, respectively *probably* and *possibly* (*small*).

**Conclusions**

This study was the first to examine the effects of different types of recovery drinks in ecological conditions of a simulated rugby seven tournament. It showed the interest to consume a recovery drink comprising soluble milk proteins and carbohydrates, compared to water, for optimizing the physical performance stability throughout the day, and for attenuating the tournament-induced increase in muscle-damages markers.

**Acknowledgements**

This research was supported by Lactalis, France.

**References**

1. Furlan N, Waldron M, Osborne M, Gray AJ: **Ecological validity and reliability of the rugby sevens simulation protocol**. *Int J Sports Physiol Perform* 2016, **11**:749-755.

2. Hopkins WG, Marshall SW, Batterham AM, Hanin J. **Progressive statistics for studies in sports medicine and exercise science**. *Med Sci Sports Exerc* 2009, **41: 3**-13.

## A40 Effects of Probiotic Supplementation on URTI and Salivary Pattern After a Marathon Race

### Edgar Tavares da Silva^1^, Samile Amorin dos Santos^1^, Aline Venticinque Caris^1^, Graziela Rosa Ravacci^2^, Ronaldo V T dos Santos^3^

#### ^1^Department of Psychobiology, Federal University of São Paulo, Brazil; ^2^Department of Gastroenterology of Medicine Faculty, USP, Brazil; ^3^ Department of Bioscience, Federal University of São Paulo, Brazil

##### **Correspondence:** Edgar Tavares da Silva (tavares.silva@unifesp.br)

**Background** Different physiological and biochemical responses are generated during and after strenuous physical exercises. These modifications can negatively impact the immune and gastrointestinal system, which consequently cause a transient immunosuppression. Different strategies are adopted to minimize the damage caused by strenuous physical exercise, such as, macro and micronutrients supplementation. Recently studies evaluate the effects of probiotics supplementation on immune function and intestinal microbiota maintenance of athletes. Scientific evidence suggests that intestinal microbiota dysfunction impact negatively on the immune response. The objective of this study was to evaluate the effect of probiotic supplementation for 30 days on the symptoms of upper respiratory tract infections in athletes and salivary pattern after a marathon.

**Materials and Methods**

Fourteen marathoners were supplemented with probiotics with 10 x 10 ^9^ CFU of *Lactobacillus Acidophilus, Lactobacillus Casei, Lactobacillus Lactis, Bifidobacterium Lactis and Bifidobacterium Bifidum* (n = 07) or placebo -corn starch (n = 07) for thirty days. The study was double blind and controlled by placebo. Before the period of supplementation, 24 hours before the race, immediately after and 1 hour after the race were collected saliva for determination of salivary pattern and application of questionnaires for seven days for analysis of respiratory symptoms. The normality of the data was checked by Shapiro-Wilk test's, from the normal distribution of the data was applied two-way Anova with posthoc Tukey’s test and significance level adopted was of p ≤ 5%.

**Results**

Our results showed that probiotic supplementation is associated with a lower amount and severity of symptoms, with less recovery time and less URTI when compared with placebo group. In the first two days there was a higher number of symptoms in the group supplemented when compared to placebo p ≤ 5%. However, after the third day there was no significant difference. Besides that, the probiotic group after the fifth day did not show any symptoms, while in the placebo group was verified significant increase p ≤ 5% of URTS. Regarding salivary parameters, no significant differences were found between the groups in the concentration of IgA, salivary flow and the rate of salivary secretion.

**Conclusions**

Our results corroborate with the recent literature, demonstrating that 30 days of supplementation with probiotics is able to mitigate the immunodepression generated by strenuous exercise without, however, having been found significant changes in salivary parameters, that classically are singled out as immunoprotective of the mucosa and upper respiratory tract. Thus, we suggest that the immunomodulatory effect of probiotics is independent of the immunity of the oral mucosa.

**Acknowledgements**

Financial Support: FAPESP #2016/25821-5 and we declare that there is no conflict of interest in research.

## A41 Six Weeks of Pyrroloquinoline Quinone Supplementation with Aerobic Training Does Not Differentially Improve Aerobic Performance Adaptations Within Untrained Young Males

### Paul S. Hwang^1^, Steven B. Machek^1^, Thomas D. Cardaci^1^, Caelin Kim^1^, Dylan T. Wilburn^1^, Emiliya S. Suezaki^1^, & Darryn S. Willoughby^1^, FISSN

#### ^1^Exercise & Biochemical Nutrition Laboratory, Department of Health, Human Performance, & Recreation, Robbins College of Health and Human Sciences, Baylor University, Waco, TX, USA

##### **Correspondence:** Darryn S. Willoughby (darryn_willoughby@baylor.edu)

**Background**

Aerobic training (ET) is known to elicit intramuscular adaptations leading to elevations in mitochondrial biogenesis, oxidative capacity, and mitochondrial function. Pyrroloquinoline quinone (PQQ) is a novel supplement involved in physiological properties including redox modulation, cellular energy metabolism, and mitochondrial biogenesis. Since aerobic exercise and PQQ exhibit similar mechanisms underlying mitochondrial adaptations, it is plausible that PQQ may carry ergogenic potential. However, no data exists surrounding PQQ supplementation with exercise in humans. Therefore, the purpose of this study was to examine the effects of a 6-week ET program in conjunction with PQQ or placebo (PLC) supplementation on aerobic performance in young untrained males.

**Materials and Methods**

In a randomized, double-blind, placebo-controlled design, untrained [<3 hr/wk exercise for > 1 year prior to starting the study] males between the ages of 18-35 (n=23) were randomly assigned to ingest 20mg/day of encapsulated PQQ (n=12) or cellulose placebo (PLC; n=11) while participating in a supervised 6-week ET program (5d/wk). Participants completed 2 research visits (baseline and 6 weeks post-ET) and aerobic performance markers were assessed. Participants ingested their respective supplement prior to a VO_2_peak test on a stationary bike. Aerobic performance variables included VO_2_peak, respiratory exchange ratio (RER), ventilatory threshold (VT), maximal heart rate (HRmax), ventilation (VE), VT percentage of VO_2_peak (VTpercVO_2_Peak), and total test time (TT). Factorial 2x2 Supplement [PQQ/PLC] by Time [Pre/Post] ANOVA with repeated measures was conducted for all criterion variables with a significance level of p<0.05.

**Results**

There were no significant supplement by time interactions for all performance variables (p>0.05). However, 6 weeks of ET enabled significant improvements in VO2peak (p<0.001), VT (p<0.001), VTpercVO2Peak (p=0.003), VE (p<0.001), and TT (p<0.001). Additionally, the PQQ group had a lower overall RER compared to PLC (p=0.033). Although not statistically significant, 6 weeks of ET presented a higher mean improvement in VTpercVO_2_peak for the PQQ group (9.425%) versus PLC (4.551%) (p=.256)

**Conclusions**

The current study suggests PQQ supplementation with 6 weeks of ET does not facilitate superior improvements in aerobic performance within untrained males over ET alone. However, the lower overall RER and higher mean improvements in VTpercVO_2_peak within the PQQ group suggests possible improvements in aerobic efficiency. Additional research is needed to examine the extent to which these adaptations are underlined by PQQ’s role to enhance the mitochondrion at the molecular level. Furthermore, future research should explore whether variations in training status, duration, modality or intensity of exercise may elicit favorable ergogenic benefits with PQQ supplementation.

## A42 Acute oral ingestion of citrulline-malate does not enhance cycling performance in recreationally active individuals

### Joshua L Gills^1^, Blake Spliker^1^, Abigail Groos^1^, Jeffery Rogers^1^, Jordan M Glenn^1,2^, Michelle Gray^1^

#### ^1^University of Arkansas, Fayetteville, AR, USA; ^2^Neurotrack Technologies Inc., Redwood City, CA, USA

##### **Correspondence:** Joshua L Gills (jgills@uark.edu)

**Background**

Citrulline-Malate (CM) purportedly increases performance through upregulating nitric oxide production (NO), which augments vasodilatory properties. Increased vasodilation leads to greater blood flow and oxygen delivery to working muscles during exercise. During races, cyclists regularly transition from aerobic to anaerobic states as they sprint towards the finish line. However, while several studies have investigated CM supplementation on cycling performance, none have examined this concept of performance benefits during aerobic cycling with subsequent transition to anaerobic cycling performance. Therefore, the purpose of this study was to examine effects of acute CM supplementation on aerobic cycling capacity and immediate subsequent anaerobic cycling performance in recreationally active males and females.

**Materials and Methods**

44 recreationally active (*n* = 31 males, *n* =13 females; Vo_2peak_ = 51.1 ± 10.8 ml•kg^-1^•min^-1^) subjects (62.0 ± 25.3 kg; 148.4 ± 57.1 cm, 20.8 ± 4.8 years) completed 2 randomized, crossover, double-blind trials consuming CM (8 g dextrose + 8 g CM) or a placebo (8 g dextrose). Prior to supplementation visits, subjects completed a Vo_2peak_ test and were familiarized to the cycling protocols on separate days. During supplementation trials, participants performed an aerobic time-to-exhaustion cycling protocol (TTE), followed by an immediate 30-second Wingate cycling test. To control for hormonal regulation, females were required to be menstruating during each intervention trial. A 1-week washout was required between trials. No alcohol, caffeine, or vigorous exercise was permitted 24-hours before trials and no food or drink intake was permitted up to 3 hours before trials.

**Results**

No significant differences in TTE performance time (p = .97) or TWC (p = .87) were observed between supplementation trials. Similar non-significant results were observed when transitioning to immediate anaerobic cycling performance on the Wingate for mean watts (p = .82), peak watts (p = .25), and anaerobic capacity (p = .99). A repeated-measures ANOVA revealed no significant difference between trials for fatigue (p = .65). Finally, independent of supplementation, significant differences (p > .01) were observed between sexes during TTE and Wingate performance for all variables.

**Conclusions**

There is natural transition from an aerobic to an anaerobic state as races commence (i.e. riders sprinting to the finish). However, in this study, CM did not increase performance in either sex as compared to the placebo. Future investigation should evaluate other supplementation options to improve this transition point as it is a critical component of competition.

## A43 The effects of varying levels of engagement on resting metabolic rate and cognitive performance metrics in college aged women

### Edward H. Robinson IV^1^ (ehrobinson@meredith.edu)

#### ^1^ Exercise and Sports Science, Department of Nutrition, Health, and Human Performance, Meredith College, 3800 Hillsborough St, Raleigh, NC, USA

**Background**

Nutrition and supplementation are often utilized in research involving cognitive performance and energy utilization. Determining energy need in nutritional research is often an important component to insure a proper supplementation or nutritional prescription. Components of cognitive performance often associated with supplementation and physical activity include measures of awareness, stress, and focus. Sequestering participants, sometimes for several hours [1], in a quiet environment to insure that cognitive measures are consistent and a true resting metabolic rate is achieved is standard practice. To date, there has been little research to determine a threshold level of engagement which might impact cerebral function or resting metabolic rate with regard to this practice of isolation [2].

**Materials and Methods**

Twenty-three healthy women (Age: 19.3±0.1yrs; Weight; 68.87±9.7Kg; Height: 162.89±9cm) volunteered to participate in this study. Resting metabolic rate was measured in one 60 minute, crossover design session measuring VO_2_ and electrical activity of the brain with four randomly assigned 15 minute measurement periods where individuals either relaxed with no external stimulation (BAS), were allowed to play a self-selected playlist of music (MUS), were allowed to use technology reading social media—with no sound or video (SOC), or were asked to play a video game (GAM). Outcomes were measured utilizing a repeated measures ANOVA.

**Results**

Repeated measures ANOVA with a Greenhouse-Geisser correction determined a significant difference observed for metabolic measurement (*F*(2.511,0.339)=3.366, *p*=0.032). Post hoc analysis revealed significance only between BAS and GAM (BAS: 3.07±0.1ml/kg/min; GAM: 3.3±0.11ml/kg/min, *p*=0.022). Significant differences were also seen for measures of awareness (*F*(2.014, 282.09)=12.51, *p*<0.001) with post hoc revealing significance between BAS and all other testing conditions(BAS: 26.12±0.35au; SOC: 31.86±1.243au, *p*<0.001; MUS: 32.46±1au, *p*<0.001; GAM: 29.63±0.82au, *p*=0.003). No significant differences were seen for stress metric (*F*(1.516, 106.6)=1.541, *p*=0.229) A significant difference was also observed for focus metric(F(2.28,507.6)=21.28, *p*<0.001). Post hoc analysis found significance only between BAS and GAM conditions (BAS: 41.65±0.9au; GAM: 50.6±1.81au, *p*<0.001).

**Conclusions**

These findings suggest that self-selected music or reading social media do not alter RMR or focus in college aged women. Higher level engagement, gameplay, did result in increased metabolic activity and heightened state of focus. While stress was not affected be increased engagement, arousal was increased above BAS in every category. The common practice of isolating or restricting individuals from all external stimuli during testing may not be necessary to obtain a true resting metabolic rate however, may still be advised if maintenance of basal cognitive performance is necessary.

**References**

1. Gonzalez A, Hoffman J, Wells A, Mangine G, Townsend J, Jajtner A, Wang R, Miramonti A, Pruna G, LaMonica M, Bohner J. Effects of time-release caffeine containing supplement on metabolic rate, glycerol concentration and performance. Journal of sports science & medicine. 2015 Jun;14(2):322.
2. Compher C, Frankenfield D, Keim N, Roth-Yousey L. Best practice methods to apply to measurement of resting metabolic rate in adults: a systematic review. Journal of the American Dietetic Association. 2006 Jun 1;106(6):881-903.

## A44 An optimized method for determining skeletal muscle sarcoplasmic and myofibrillar protein concentrations

### Carlton D. Fox^1^, Kaelin C. Young^1,2^, Christopher G. Vann^1^, Paul A. Roberson^1^, Shelby C. Osburn^1^, Johnathon H. Moore^1^, Petey W. Mumford^1^, Matthew A. Romero^1^, Darren T. Beck^1,2^, Cody T. Haun^3^, Andreas N. Kavazis^1,2^, Michael D. Roberts^1,2^

#### ^1^School of Kinesiology, Auburn University, Auburn, AL USA; ^2^Department of Cell Biology and Physiology, Edward Via College of Osteopathic Medicine - Auburn Campus, Auburn, AL USA; ^3^LaGrange College, LaGrange, GA USA

##### **Correspondence:** Carlton D. Fox (cdf0007@auburn.edu)

**Background**

Several researchers have sought to isolate skeletal muscle myofibrillar protein for downstream assays. While various published methods exist, no study to date has thoroughly compared how these methods differ in protein yield and fidelity.

**Materials and Methods**

Herein, five different methods were used to isolate myofibrillar proteins from 20-25 mg gastrocnemius muscles of six male Fischer rats. These methods included: a) the guanidinium thiocyanate-phenol-chloroform extraction (Trizol) method, b) a general cell lysis (GCL) method using a 20 mM Tris/150 mM NaCl/1.0% Triton-X 100 buffer, c) a two-step method which included 20 mM Tris/100 mM KCl/5 mM EGTA/1.0%Triton-X 100 (step 1) + 20 mM Tris/500 mM KCl/20% glycerol (step 2) (MF method 1), d) MF method 1 modified to include 50 mM spermidine in step 2 (MF method 2), and e) a two-step method which included 20 mM Tris/0.5% Triton-X 100 (step 1) + 0.3 M sodium hydroxide (step 2) (MF method 3). Following isolation protocols, total myofibrillar protein concentrations were assessed using colorimetric spectrophotometry. The fidelity of myofibrillar proteins was also assessed using SDS-PAGE followed by Coomassie staining as well as myosin and actin immunoblotting.

**Results**

MF methods 2 & 3 yielded greater myofibrillar protein concentrations compared to the other methods (p<0.05). SDS-PAGE with Coomassie staining of myofibrillar fractions from each method indicated: a) MF methods 1 & 2 yielded thick bands at 43 kD and 220 kD; the molecular weights of actin and myosin, respectively, b) MF method 3 yielded a whole-lane smear which is likely due to alkaline-mediated hydrolysis, c) GCL yielded a thick band at 43 kD, but a faint to non-existent band at 220 kD, and d) the Trizol method yielded virtually no bands. Western blotting was not possible on MF method 3 samples, but MF method 2 yielded significantly more actin and myosin compared to the Trizol and GCL methods (p<0.05). MF methods 1-3 are the only methods that yielded a sarcoplasmic fraction and while Coomassie staining indicated that the lane profiles of these methods were similar, Western blotting indicated that MF method 3 yields a sarcoplasmic fraction with the least amount of myosin contamination.

**Conclusions**

Through thorough comparative analyses we have identified methods which isolate high fidelity sarcoplasmic and myofibrillar protein fractions suitable for total protein quantification, Western blotting, and proteomic analysis.

## A45 Evaluating the Effects of Hormonal Contraceptive Use on Changes in Biomarkers During the Competitive Season in DI NCAA Female Soccer Players

### Brittany N. Bozzini^1^, Bridget A. McFadden^1^, Alan J. Walker^1^, Michelle A. Arent^1^, and Shawn M. Arent, FISSN^1,2^

#### ^1^IFNH Center for Health and Human Performance, Rutgers University, New Brunswick, NJ, USA; ^2^Department of Kinesiology and Health, Rutgers University, New Brunswick, NJ, USA

##### **Correspondence:** Shawn M. Arent (shawn.arent@rutgers.edu)

**Background**

The high training load throughout the competitive season in DI NCAA soccer has been shown to induce changes in biomarkers in female athletes including markers of stress, inflammation, and reproductive function. Additionally, hormonal contraceptive (HC) use has been observed to cause elevations in cortisol and c-reactive protein and thus, changes in these biomarkers may be exacerbated in athletes using HCs. Therefore, the purpose of this study was to compare biomarkers changes in female soccer players with and without HC use throughout the competitive season.

**Materials and Methods**

Female collegiate soccer players were stratified into two groups (HC and Control [CON]) based on their reported HC use at the start of season (HC: N=10, M_age_=19.1±0.9yrs, M_BF%_=20.0±6.7, M_VO2MAX_=47.9±3.4ml/kg/min; CON: N=16, M_age_=18.9±1.1yrs, M_BF%_=19.9±4.7kg, M_VO2MAX_ =49.6±4.5ml/kg/min). Blood draws were performed prior to preseason as well as weeks 2, 4, 8, & 12 of the season. Athletes arrived between 0700-0900h, 18-24 hours post-game in a fasted, euhydrated state. Total cortisol (TCORT), free cortisol (FCORT), c-reactive protein (CRP), interleukin-6 (IL-6), estradiol (E_2_), sex-hormone binding globulin (SHBG), follicle-stimulating hormone (FSH), progesterone (P_4_), total testosterone (TTEST), and free testosterone (FTEST) were analyzed. For each biomarker, area under the curve was calculated across all blood draw timepoints and ANOVAs were conducted with significance set at p<0.05.

**Results**

Female players on HCs had significantly greater TCORT (p<0.01) and CRP (p<0.05) throughout the competitive season than CON. Trends for greater SHBG (p=0.06) and decreased FTEST (p=0.08) in the HC group were also observed. No differences were depicted between groups for all other biomarkers throughout the season including FCORT, E_2_, FSH, IL-6, P_4_, and TTEST (p>0.05). Secondary subgroup analysis revealed that those on oral contraceptives (N=6) had significantly greater TCORT (p<0.05) and trends for lower FSH (p=0.07) and increased leptin (p=0.08) than those with intrauterine devices (N=4).

**Conclusion**

These results support previous research that has associated HC use with increased levels of cortisol, CRP, and SHBG and reductions in testosterone. The increased levels of stress and inflammatory biomarkers in the female players using HCs beyond those experienced in the CON may be indicative of an exacerbated catabolic environment in these athletes. This heightened biomarker response due to the combination of high training load and HC use may also denote increased recovery needs in HC athletes. Further research examining the effects of different types of HCs in a larger population on the stress response and the implications on performance and recovery appears warranted.

**Acknowledgments**

Funding provided by Quest Diagnostics

## A46 Validity of Different Skinfold Equations to Calculate Body Fat Percentage from Ultrasound Measures in High-Level, Adolescent Dancers

### Harry P. Cintineo^1^, Alexa J. Chandler^1^, David J. Sanders^1^, Alan J. Walker^1^, Bridget A. McFadden^1^, Brittany N. Bozzini^1^, Marissa L. Bello^1^, Morgan S. Murray^1^, Robert Monaco^2^, Shawn M. Arent, FISSN^1,3^

#### ^1^IFNH Center for Health and Human Performance, Rutgers University, New Brunswick, NJ 08901, USA; ^2^Atlantic Health System, Morristown, NJ 07960, USA; ^3^Department of Kinesiology and Health, Rutgers, University, NJ 08901, USA

##### **Correspondence:** Shawn M. Arent (shawn.arent@rutgers.edu)

**Background**

Body composition is a critical aspect of athlete health and performance, especially in sports with an aesthetic component. Assessment that is time-efficient, easy-to-use, portable, and affordable is ideal in this population. Brightness-mode ultrasound (B-US) offers these features, particularly when measuring a minimal number of sites. The purpose of this study was to compare 7-site, 4-site, and 3-site modified Jackson-Pollock equations using air displacement plethysmography (ADP) as reference.

**Materials and Methods**

Male (N=21; M_age_=17.0±1.7 y; M_weight_=62.4±10.4 kg) and female (N=28; M_age_=15.6±1.3 y; M_weight_=47.8±4.6 kg) dancers arrived at the laboratory having refrained from food and water for ≥2 hours. Body fat percentage (%BF) was assessed using ADP. Next, subcutaneous adipose tissue (SAT) was measured using B-US at seven sites: pectoralis, subscapula, triceps, midaxilla, suprailiac, abdomen, and thigh. %BF was calculated using modified Jackson-Pollock 7- (7BUS), 4- (4BUS), and 3-site (3BUS) skinfold-thickness (SKF) equations. Pearson product correlations quantified associations between variables, and paired-samples T-tests assessed differences between measures. Significance was set at P<0.05.

**Results**

Significant correlations were found between ADP and 7BUS, 4BUS, and 3BUS across all subjects (r>0.86; P<0.01) and in females (r>0.85; P<0.01). In males, the strongest correlation with ADP was 7BUS (r=0.771; P<0.01), while 3BUS (r=0.730; P<0.01) and 4BUS (r=0.488; P=0.025) were weaker. All B-US %BF values correlated with one another in all subjects (r>0.96; P<0.01). In females, ADP was significantly lower than 7BUS and 3BUS (p<0.01) and approached significance for 4BUS (p=0.081). However, only 3BUS was significantly higher than ADP in males (P<0.01). In the entire sample and females only, 7BUS was significantly higher than 4BUS and significantly lower than 3BUS (P<0.01); 4BUS was significantly lower than 3BUS (P<0.01). Males followed this pattern, except the difference between 7BUS and 4BUS was non-significant (P=0.236).

**Conclusions**

These findings support the use of 7-, 4-, and 3-site BUS in adolescent female dancers. Although the highest correlation with ADP was seen with 7-site, 3-site BUS may be the most time-efficient method in these individuals. Despite non-significant differences between ADP, 7BUS, and 4BUS in males, weaker correlations suggest that this method of assessing %BF may be inappropriate. These results may be explained by less SAT in males compared to females. Thus, differentiation between tissues and overall interpretation of BUS may be more prone to experimenter error, particularly in leaner individuals. Despite strong correlations, significant differences between 7BUS, 4BUS, and 3BUS further support the idea that SKF equations may not be applicable to US, and US-specific equations should be developed.

## A47 The Effect of a Collegiate Preseason on Energy Status and Biomarkers in Women’s Division 1 Soccer Players

### Bridget A. McFadden^1^, Brittany N. Bozzini^1^, Alan J. Walker^1^, David J. Sanders^1^, Christopher E. Ordway^1^, Michelle A. Arent^1^, Shawn M. Arent, FISSN^1,2^

#### ^1^ IFNH Center for Health and Human Performance Rutgers University, New Brunswick, NJ, USA ; ^2^Department of Kinesiology and Health Rutgers University, New Brunswick, NJ, USA

##### **Correspondence:** Shawn M. Arent (shawn.arent@rutgers.edu)

**Background**

Relative Energy Deficiency in Sport is characterized by inadequate energy intake (EI) to support various body functions essential for optimal health and performance. Energy availability (EA) considers EI in relation to exercise energy expenditure (EEE). Methods that readily detect energy status are currently lacking, yet biomarkers show promise in detecting physiological changes due to training and nutrition. The purpose of this study was to assess EA in conjunction with body composition and biomarker changes in female soccer players during preseason.

**Materials and Methods**

Women’s DI college soccer players (N=26; M_weight_=65.78±6.5kg) were monitored throughout the two-week preseason. Body composition was assessed via Bodpod prior to the start of preseason (T1) and immediately after preseason (T2) to determine fat free mass (FFM) and percent body fat (%BF). Blood draws were performed at T1 and T2. Athletes arrived between 0700-0900h in a fasted and euhydrated state. Total cortisol (CORT), growth hormone (GH), insulin-like growth factor-1 (IGF-1), prolactin (PRL), adiponectin (ADIP), leptin (LEP), triiodothyronine (T_3_), thyroxine (T_4_), and thyroid-stimulating hormone (TSH) were analyzed. Players were monitored during all practices using the Polar TeamPro system to determine EEE. Caloric intake was tracked via 3-day (Thursday-Saturday) dietary food logs. EA was calculated using the formula (EI_AVG_-EEE_AVG_/FFM). Players were first grouped into “low” EA (<30 kcal/FFM; n =17) and “normal” EA (>30 kcal/FFM, n=9). A secondary analysis was used to stratify players using >40 kcal/FFM (n=10), 25-40 kcal/FFM (n=12), and <25 kcal/FFM (n=4) cutoffs. RM MANOVAs with univariate follow-ups were conducted with significance set at P<0.05.

**Results**

A significant time effect was seen for an increase in FFM from T1 to T2 (P<0.05), however there were no differences between groups for %BF or FFM (P>0.05). Decreases were seen for TSH (P<0.05) with trends for increases in GH (P=0.099) and decreases in PRL (P=0.087) from T1 to T2. No differences between groups were seen for any biomarkers analyzed (P>0.05). Further, in the secondary analysis no group differences were seen for any biomarkers (P>0.05).

**Conclusion**

The time effects seen for select biomarkers indicates measurable changes during times of high EEE over a short, condensed pre-season. However, no differences in biomarkers were seen between EA groups. Further, increases in FFM during preseason were observed across groups despite only 4 players falling into the “optimal” EA category. This indicates a need for studies evaluating macronutrient content and energy status as well as the effect of EA on biomarkers and body composition over a longer period.

**Acknowledgements**

Funding supported by Quest Diagnostics

## A48 Changes in Nutritional Biomarkers and Performance in High-Level Youth Dancers Over Time

### Marissa L. Bello^1^, David J. Sanders^1^, Alan J. Walker^1^, Bridget A. McFadden^1^, Harry P. Cintineo^1^, Brittany N. Bozzini^1^, Morgan S. Murray^1^, William G. Maldonado^1^, Michelle A. Arent^1^, Shawn M. Arent FISSN^1,2^

#### ^1^IFNH Center for Health and Human Performance, Rutgers University, New Brunswick, NJ, USA; ^2^Department of Kinesiology and Health, Rutgers University, New Brunswick, NJ, USA

##### **Correspondence:** Shawn M. Arent (shawn.arent@rutgers.edu)

**Background**

Balancing training and recovery is crucial for athletic performance in individuals of all ages. Proper nutrition plays a large role in facilitating recovery and optimizing performance; however, little research exists in adolescent dancers. The purpose of this study was to observe changes in nutritional biomarkers and performance in high-level youth dancers throughout the performance season.

**Materials and Methods**

High-level youth dancers (Males: N=10, M_age_=16.36±1.6yrs, M_weight_=62.12±10.07kg; Females: N=14, M_age_=15.43±1.3yrs, M_weight_=47.86±4.71kg) participated in blood draws prior to the performance season (T1) and every subsequent four weeks (T2-T5). Iron (Fe), total iron binding capacity (TIBC), percent saturation of iron (%Sat), omega-3 fatty acids (OMG3), omega-6:omega-3 ratio (OMG63), vitamin B_12_ (VitB_12_), folate, and vitamin D (VitD) were analyzed. Performance testing was conducted pre- and post-season to assess body composition via BodPod, vertical jump (VJ), and aerobic fitness (VO_2max_). RM-ANOVAs with univariate follow-ups were conducted to analyze changes in biomarkers and performance as a function of sex as well as changes within each sex. Significance was set at P<0.05.

**Results**

There was a significant sex-by-time interaction for %Sat (P=0.027) and a trend for Fe (P=0.056). No significant differences between sexes were observed for any other biomarkers (P>0.05). Over the course of the study, there were significant increases in males with %Sat (P<0.05), and decreases in VitB_12_ at T3 and T5 with an increase at T4 (P<0.05). Females experienced significant decreases in Fe and %Sat (P<0.05), and changes in folate similar to the pattern of VitB_12_ in the males (P<0.05). VJ significantly increased over the season in males (P<0.05) with no significant change in females. No significant changes in body composition or VO_2max_ were seen over time for either sex (P>0.05).

**Conclusions**

The changes in biomarkers may indicate alterations in nutritional status in youth dancers over the course of the performing season. The sex-by-time interactions for Fe and %Sat suggest females may be at greater risk for Fe deficiency compared to males. Despite non-significant changes in VitD, it is important to note that all values remained below clinical ranges and were categorized as insufficient. Thus, supplementation with VitD in this population as well as Fe for females may be beneficial, especially during times of high-volume training. Overall, despite fluctuations in nutritional biomarkers, body composition and performance were not adversely affected between pre- and post-testing. Further research in a larger sample of youth dancers is warranted to support these findings for nutritional supplementation and its implications on performance.

**Acknowledgments**

Funding provided by Quest Diagnostics.

## A49 Examining the Effects of a Mid-Season Supplement Intervention on Anabolic and Catabolic Biomarkers in Division I NCAA Female Soccer Players

### Traci A McCarthy^1^, Bridget A. McFadden^1^, Brittany N. Bozzini^1^, Alan J. Walker^1^, Michelle A. Arent^1^, Shawn M. Arent, FISSN^1,2^

#### ^1^IFNH Center for Health and Human Performance Rutgers University, New Brunswick, NJ, USA; ^2^Department of Kinesiology and Health Rutgers University, New Brunswick, NJ, USA

##### **Correspondence:** Shawn M. Arent (shawn.arent@rutgers.edu)

**Background**

Collegiate athletes are faced with a stressful season marked with high training loads, reduced recovery, and altered dietary practices. Adequate nutrition is essential to promote recovery and maintain performance throughout the season. The purpose of this study was to examine the effects of a mid-season intervention of a protein or carbohydrate supplement on anabolic and catabolic biomarkers throughout a DI NCAA female soccer season.

**Materials and Methods**

Collegiate female soccer players (N=30; M_Age_=19.3±1.1yrs) participated in blood draws prior to the start of preseason (T1) and every 28-days throughout the season (T2-T4). Athletes arrived fasted and euhydrated between 0700-0900h for blood draws, 18-24 hours post-game. Growth hormone (GH), insulin-like growth factor-1 (IGF-1), free cortisol (FCORT), total cortisol (TCORT), progesterone (PRG), interleukin-6 (IL-6), prolactin (PRL), and tumor necrosis factor-alpha (TNF-α) were assessed. Supplement intervention occurred at the midpoint of the season (T3-T4). Subjects were randomly assigned to either 40g/day of whey protein (PRO; N=6) or carbohydrate (CHO; N=8) supplementation to be taken daily in addition to their normal diets. Subjects who adhered to supplementation for <20% of the study were used as controls (CON; N=9). RM-MANOVAs with univariate follow-ups were conducted with significance set at P<0.05.

**Results**

There was a significant time main effect for an increase in GH and FCORT and decreases in TNF-α and IGF-1 fromT1-T3 (P<0.05) along with a trend for increased PRG (p=.091). There were no significant group differences found for any biomarkers prior to the supplementation intervention. Significant time effects were seen for decreases in TCORT (P<0.05) during the intervention (T3-T4). No significant differences in biomarkers were observed between groups post-intervention (P>0.05), however a trend for a time-by-group interaction was seen for IL-6 (P=0.077). Graphic trends indicated modest benefits of PRO for GH, cortisol, TNF-α and IL-6.

**Conclusion**

The fluctuations in biomarkers observed throughout the season may be reflective of the accumulated stress of the season. Dietary supplementation may help offset these changes along with adequate recovery. Despite the lack of differences between groups, the pattern observed for PRO may suggest that the macronutrient, not just supplemental calories, may be an important consideration to combat the stress of the season. Further research examining supplementation in a larger population over a longer time period may provide insight into the preferred macronutrient for enhanced recovery. Additionally, classifying the nutritional status of the athlete may help further clarify impact.

**Acknowledgements:** funding supported by Quest Diagnostics with special thanks to Milk Specialty Global, Dymatize Nutrition, and Banned Substance Control Group (BSCG)

## A50 The Validity of Different Ultrasound Devices and BIA to Assess Body Fat Percentage in Adolescent Dancers

### Alexa J. Chandler^1^, David J. Sanders^1^, Harry P. Cintineo^1^, Morgan S. Murray^1^, Brittany N. Bozzini^1^, Marissa L. Bello^1^, Alan J. Walker^1^, Bridget A. McFadden^1^, Robert Monaco^2^, Shawn M. Arent, FISSN^1,3^

#### ^1^ IFNH Center for Health and Human Performance Rutgers University, New Brunswick, NJ, 08901, USA; ^2^ Atlantic Health System, Morristown, NJ, 07960, USA; ^3^ Department of Kinesiology and Health Rutgers University, New Brunswick, NJ, 08901, USA

##### **Correspondence:** Shawn M. Arent (shawn.arent@rutgers.edu)

**Background**

Amplitude- (AUS) and brightness-mode (BUS) ultrasound are inexpensive, portable body-fat percentage (%BF) assessment devices. However, research with these tools is limited in contrast to other laboratory-based techniques (e.g. air displacement plethysmography (ADP), bioelectrical impedance (BIA)). Therefore, the purpose of this study was to evaluate the validity of %BF assessment via AUS, BUS, and BIA compared to ADP.

**Materials and Methods**

Twenty adolescent dancers (M_age_=16.8±1.7; M_BMI_=19.5±1.9) performed %BF testing via ADP, BIA, AUS, and BUS. Subjects were measured at 7-sites using AUS and BUS. %BF, fat-free mass (FFM) and fat-mass (FM) were determined using modified Jackson-Pollock equations. Pearson product moment correlations were used to determine relationships between measures. Dependent t-tests were performed to analyze differences between methods in the total sample and each sex (males: n=7; females: n=13). Significance was set at P<0.05.

**Results**

Across all subjects, %BF and FFM were strongly correlated with ADP for all devices (BUS_%BF_ r=0.908; AUS_%BF_ r= 0.887; BIA_%BF_ r=0.879; BUS_FFM_ r=0.994; AUS_FFM_ r=0.989; BIA_FFM_ r=0.980; P<0.05). In females, %BF and FFM from all devices was strongly correlated with ADP (BUS_%BF_ r=0.947; AUS_%BF_ r=0.926; BIA_%BF_ r=0.905; BUS_FFM_ r=0.957; AUS_FFM_ r=0.907; BIA_FFM_ r=0.977; P<0.05). In males, ADP_%BF_ showed a moderately strong correlation to AUS_%BF_ (r=0.757; P<0.05), and a trend for BUS_%BF_ (r=0.722, P=0.068). However, ADP_FFM_ was strongly correlated with all devices for males (BUS_FFM_ r=0.990; AUS_FFM_ r=0.980; BIA_FFM_ r=0.910). Dependent t-tests revealed trends towards significantly higher BIA_%BF_ compared to ADP_%BF_ (P=0.073), but no differences between ADP_%BF_ and AUS_%BF_ (P=0.949) or BUS_%BF_ (P=0.199). In females, ADP_FFM_ was significantly higher than BUS_FFM_ (P=0.024), and higher ADP_%BF_ than BUS_%BF_ trended towards significance (P=0.071). There were no other differences in %BF or FFM for either sex.

**Conclusions**

Ultrasound and BIA appear to be valid %BF tools in adolescent female, but not male, dancers. Stronger correlations between FFM, compared to %BF, via BIA and US in both sexes may indicate that FFM is a more reliable measure than %BF using these devices in youth athletes. This may be due to a combination of small differences in FM and FFM between devices, causing significant %BF differences, especially in very lean athletes. However, ultrasound may have increased benefits over ADP and BIA because of the ability to provide regional FFM analysis, yielding more precise measures during maturation. Sex-specific differences between devices needs further investigation, as there were significant differences between ADP_FFM_ and BUS, despite strong correlations, in females, and weaker correlations for all variables in males compared to females.

## A51 Changes in Stress-Related Biomarkers in High-Level Youth Dancers Over a Performance Season

### David J. Sanders^1^, Alexa J. Chandler^1^_,_ Marissa L. Bello^1^, Alan J. Walker^1^, Bridget A. McFadden^1^, Harry P. Cintineo^1^, Brittany N. Bozzini^1^, Morgan S. Murray^1^, Michelle A. Arent^1^, Shawn M. Arent FISSN^1,2^

#### ^1^IFNH Center for Health and Human Performance, Rutgers University, New Brunswick, NJ, USA; ^2^Department of Kinesiology and Health, Rutgers University, New Brunswick, NJ, USA

##### **Correspondence:** David J. Sanders (d.sanders@rutgers.edu)

**Background**

Biomarker monitoring enables the evaluation of an individual athlete’s physiological response to training. Currently, research in elite, adolescent artistic-athletes (e.g. dancers) is limited, particularly in regard to changes in biomarkers throughout a performance season. The purpose of this study was to assess changes in stress-related biomarkers across a performance season and to identify differences between high-level male and female ballet dancers.

**Materials and Methods**

Male (n=9; M_age_=17.2±1.2yrs; M_height_=183.5±6.4cm; M_weight_=64.5±8.7kg) and female (n=10; M_age_=15.8±14yrs; M_height_=166.1±3.9cm; M_weight_=49.7±3.3kg) high-level ballet dancers participated in blood draws prior to the beginning of the performance season (T1), and every 4-weeks thereafter (T2-T5). Total cortisol (CortT), free cortisol (CortF), total testosterone (TestT), free testosterone (TestF), estradiol (E2), follicle-stimulating hormone (FSH), progesterone (Prg), prolactin (Prl), sex-hormone binding globulin (SHBG), creatine kinase (CK), and interleukin-6 (IL-6) were assessed. RM-ANOVAs with univariate follow-ups were conducted to analyze changes in biomarkers as a function of sex as well as changes within each sex. Significance was set at P<0.05.

**Results**

A significant sex-by-time interaction was found for FSH only (P=0.036). Group main effects were found in E2, TestT, TestF, FSH, SHBG, and CK (P<0.05). Time main effects were found in CortF, CK, and IL6 (P<0.05). IL6 was significantly greater than baseline at all time points, with the largest difference at T4. In males there were significant decreases in CortF, Prg and CK from T1 to T5 (P<0.05). In females, a trend for a decrease in FSH was found from T1 to T5 (P=0.06), with a significant difference from T2 to T5 (P<0.05). Significant increases were observed in males and females for IL-6 from T1 through T5 (P<0.05). SHBG was significantly greater in males at T5 than T1 (P<0.05).

**Conclusions**

Changes in biomarkers may indicate physiological stress and/or inadequate nutrition over the course of a season. Different responses are observed between sexes, with greater disruption seen in females. The sex-by-time interaction in FSH suggests disturbance in the HPG-axis in female dancers only, as the decreases in FSH coincides with a non-significant decrease in E2. Potentially, this may lead to the observation of primary or secondary amenorrhea in young female athletes. In males, significant increases in SHBG occurred, but TestT and TestF increased non-significantly. Thus, HPG-axis function appears normal in young male dancers. Interestingly, IL-6 is highest in both sexes immediately prior to scheduled performances at T4, which may have occurred due to an increase in time spent rehearsing for the performances.

**Acknowledgments:** Funding provided by Quest Diagnostics.

## A52 The Effects of Pyrroloquinoline Quinone Supplementation with Aerobic Training on Body Composition in Untrained Males

### Emiliya S. Suezaki, Paul S. Hwang, Steven B. Machek, Thomas D. Cardaci, Caelin Kim, Dylan T. Wilburn, & Darryn S. Willoughby, FISSN

#### ^1^Exercise & Biochemical Nutrition Laboratory, Department of Health, Human Performance, & Recreation, Robbins College of Health and Human Sciences, Baylor University, Waco, TX, USA

##### **Correspondence:** Darryn S. Willoughby (darryn_willoughby@baylor.edu)

**Background**

Pyrroloquinoline quinone (PQQ) is a novel supplement associated with physiological processes pertaining to improvement of mitochondria-mediated energy metabolism. As an exercise mimetic, PQQ combined with aerobic exercise synergistically modulates mitochondrial biogenesis, stimulating greater energy production and fat metabolism. Therefore, PQQ may potentiate higher intensities, longer durations, and greater bioenergetics, ultimately resulting in a more favourable body composition. However, there is a dearth of data regarding PQQ supplementation in conjunction with aerobic exercise and its impacts on body composition. Thus, this investigation aimed to examine body composition following PQQ supplementation in young untrained males performing a 6-week aerobic exercise training program (ET).

**Materials and Methods**

In a randomized, double-blind, placebo-controlled design, untrained [<3 hr/wk exercise for > 1 year prior to starting the study] males aged between 18-35 (n=23) were randomly assigned to ingest 20mg/day of encapsulated PQQ (n = 12) or cellulose placebo (PLC; n=11) while participating in a supervised 6-week ET program (5 d/wk). Participants completed 2 research visits (baseline and 6 weeks post-ET) and body composition was assessed. Participants ingested their respective supplement prior to a VO_2_peak test on a stationary bike. Body composition was assessed via DEXA. Body composition measures included lean mass, fat mass, and body fat percentages. Factorial 2x2 Supplement [PQQ/PLC] by Time [Pre/Post] ANOVA with repeated measures was conducted for all criterion variables at a significance of p<0.05.

**Results**

There were no significant interactions between group and time for all variables (p > 0.05). However, there was a main time effect for lean mass (p = 0.004). Pairwise comparisons revealed a greater increase in lean mass irrespective of group. There were no main time effects for fat mass (p = 0.113) or body fat percent (p = 0.073); however, there was a non-significant decrease of 1.033 kg and 0.182 kg in PQQ and PLC, respectively.

**Conclusions**

From the results, it appears that PQQ supplementation combined with six weeks of aerobic exercise does not elicit favourable improvements in body composition over PLC. Although significance was not met, the larger fat mass reductions in the PQQ group suggest potential greater improvements with a longer duration training protocol. Additionally, any improvements in body composition are perhaps due to low baseline muscle mass and novitiate training status.

## A53 Validation of a 3-dimensional body scanner against dual-energy x-ray absorptiometry and a 4-compartment body composition measurement model

### Katie R. Hirsch^1,2^, Gabrielle J. Brewer^1^, Malia M.N. Blue^1,2^, Austin M. Peterjohn^1^, Abbie E. Smith-Ryan^1,2^

#### ^1^Applied Physiology Laboratory, Department of Exercise and Sport Science, University of North Carolina, Chapel Hill, NC, USA; ^2^Human Movement Science Curriculum, Department of Allied Health Science, University of North Carolina, Chapel Hill, NC, USA

##### **Correspondence:** Abbie E. Smith-Ryan (abbsmith@email.unc.edu)

**Background**

Recently developed 3D body scanners use infrared imaging technology to create a full body model from which total and regional body composition is calculated using algorithmic prediction models. However, direct validation against multi-compartment laboratory criterions are lacking. The purpose of this study was two-fold: 1) to compare body composition measurements obtained from a 3D body scanner to measurements obtained from dual energy x-ray absorptiometry (DXA) in normal weight adults and, 2) to validate body composition measurements obtained from a 3D body scanner against a gold-standard four compartment (4C) body composition model.

**Materials and Methods**

Body composition (percent body fat [%BF], fat mass [FM], lean mass [LM]) of 67 young adults (Males=22; Females=45; Mean ± SD: Age: 20.6 ± 1.1 years; Height: 168.9 ± 9.9 cm; Weight: 64.8 ± 9.1 kg; BMI: 22.6 ± 2.0 kg·m^-2^) was measured by: 1) 360° total body 3D scan (Styku); 2) total body DXA scan (GE iDXA) and; 3) 4C model (Wang et al.) utilizing measurements of total body water from bioelectrical impedance spectroscopy, body volume from air displacement plethysmography, and bone mineral from DXA. Total error (TE) and standard error of the estimate (SEE) were used to evaluate prediction error of 3D compared to both DXA and 4C for the entire group.

**Results**

Compared to DXA, 3D %BF (Mean Difference ± SD: 0.1 ± 4.4%), FM (0.1 ± 2.9 kg), and LM (0.5 ± 3.0 kg) was not significantly different (p>0.05); prediction error was considered fair to fairly good for %BF (TE=4.4%; SEE=4.3%), FM (TE=4.4 kg; SEE=3.5 kg), and LM (TE=3.0 kg; SEE=3.0 kg). Compared to a 4C model, the 3D scan significantly over-predicted %BF (4.3 ± 5.3%) and FM (2.6 ± 3.5 kg) and under-predicted LM (5.8 ± 3.8 kg)(p<0.001); prediction error was considered poor for %BF (TE=6.7%; SEE=5.2%), FM (TE=4.4 kg; SEE=3.5 kg), and LM (TE=7.0 kg; SEE=3.7 kg).

**Conclusions**

Body composition estimates obtained from a 3D body scanner could be considered comparable to DXA estimations, with %BF estimates within ± 4% fat. However, when compared to a gold-standard 4C body composition model, the 3D body scan significantly over-predicted %BF (+6.7%) and FM (+4.4 kg), and under-predicted LM (-7.0 kg) in normal weight individuals. Further validation is needed to refine the predictive capacity and improve 3D scanner body composition estimations in normal weight individuals.

## A54 Nutritional supplement knowledge, attitudes and behaviors among collegiate athletes

### Kaila Vento, Kelli Reese, Floris Wardenaar

#### Arizona State University, Tempe, AZ, USA

##### **Correspondence:** Kaila Vento (kvento@asu.edu)

**Background**

Nutritional supplements are common in sports and have the potential benefits of enhancing performance. Student-athlete’s knowledge about supplements could influence their attitudes and use. Poor supplement knowledge could lead to consuming unsafe products and doping, negatively impacting sport eligibility and health. With more attention geared on nutritional supplements as an easy means to produce heightened performances, accessibility of these supplements increases as well as the risk of intaking ill manufactured products. Thus, the purpose of this research was to better understand athletes’ views of nutritional supplements use vs. self-reported knowledge and attitudes in NCAA Division I student-athletes.

**Materials and Methods**

Student-athletes from a south-western Division I university (65% female; age: 19.8±1.6 years-old) completed a questionnaire on nutritional supplement knowledge, attitudes, and use over the last 12 months. The supplement knowledge section of the questionnaire consisted of 12 questions taken from the validated GSNK questionnaire.

**Results**

Supplement knowledge scores averaged 27±16 percent. Perceived supplement knowledge on a five-point scale averaged 2.5±0.8 (range; 1-5). When classified as a low or high supplement attitudes vs. low-moderate-high knowledge scores, no difference was detected among categories [χ^2^(2)=0.538, *p*=0.80]. The three most reported dietary (N=138) and ergogenic supplements (N=125) within the last 12 months were multivitamin and mineral supplements (65%), single vitamins or minerals (64-63%), caffeine (63%), tart cherry (39%) and probiotics (32%). Banned supplements from the NCAA or unrecommended supplements (i.e., ephedra, tribulus terrestris, DHEA, and colostrum) were in the lower range of reported supplements (13-14%). No significant correlations between supplement knowledge and use were found. No significant relationships between attitude and knowledge towards NCAA impermissible supplements were found, except for B-alanine [χ^2^(2)=13.38, *p*=0.002] and creatine [χ^2^(2)=7.731, *p*=0.021] indicating that student-athletes reported lower use when perceived and self-reported knowledge was low.

**Conclusions**

Self-reported supplement knowledge tests scores were low as well as the perception of their own knowledge, but comparable to previous reporting in athletes. In general, supplement knowledge and perceived knowledge did not relate with use, expect certain NCAA impermissible supplements. While the use of banned or unrecommend nutritional supplements was infrequent, some student-athletes use these supplements for performance benefits despite regulations and lack of scientific-evidence.

**Acknowledgements**

The study was funded in part by the Graduate and Professional Student Association Athletic Research Grant at Arizona State University.

## A55 A comparison of bioimpedance spectroscopy and A-mode ultrasound to estimate the body composition of male physique competitors

### Guillermo Escalante^1^, Erika Arteaga^1^, Julio Mora^1^, Jessica Heredia^1^, Alexandra Khartabil^1^, Jason Hernandez^1^, Amanda Maravi^1^, Yadira Marin^1^

#### Department of Kinesiology, California State University San Bernardino, San Bernardino, CA, USA

##### **Correspondence:** Guillermo Escalante (gescalan@csusb.edu)

**Background**

Bioimpedance spectroscopy (BIS) and A-mode ultrasound (US) are commonly used to estimate body composition. BIS predicts body composition using the difference in electrical conductivity according to the physical quality of tissues; hence, alterations in tissue water and electrolyte content may alter electrical conductivity and create an error in the body composition estimation. Conversely, US sends ultrasound waves in tissue and strong reflections occur at the boundary of different tissue types such as fat-muscle. The reflected signal is recorded and used to estimate the subcutaneous fat thickness at various sites and hydration levels have minimal impact on the body composition estimation. Since physique competitors have been reported to implement dehydration practices prior to a competition, BIS body composition estimations may not be accurate in this population one day prior to competing. This study assessed the hydration status of physique competitors one day prior to competing and compared the BIS and US body composition estimations.

**Materials and Methods**

Ten amateur male physique competitors (age = 30.0 +/- 5.8 yrs, height = 1.72 +/- .08 m, weight = 76.4 +/- 7.6 kg) volunteered for this study. Height and weight were measured with an electronic scale and a standard stadiometer. Body fat was estimated with a BIS device (SFB7, ImpediMed, Carlsbad, CA, USA) and a US device (BodyMetrix, IntelaMetrix, Brentwood, CA, USA) per the manufacturer recommended guidelines. The Jackson-Pollock 3-site formula was used to estimate body composition based on the body density prediction equation from the subcutaneous fat measurements of the chest, abdomen, and thigh as determined by the US device. Participants also provided a urine specimen to determine the specific gravity of urine using a refractometer. A paired t-test was used to compare the body fat percentage results for each participant as estimated by the BIS and US.

**Results**

Body fat percentage measures for the BIS and US were 14.2 ± 5.1 and 7.2 ± 2.4, respectively. The t-test revealed there was a statistically significant difference in body composition between the devices (p = 0.003). The mean SG for the participants was 1.019 ± 0.011, indicating mild dehydration.

**Conclusions**

BIS may not be an ideal tool to use to measure body composition when individuals are not in a state of euhydration as is the case in physique competitors one day prior to competition. Measuring SG prior to using BIS to ensure euhydration may be a good practice to implement to maximize accuracy.

## A56 Resistance and cardiovascular training practices of in-season male physique competitors

### Guillermo Escalante, Erika Arteaga, Jessica Heredia, Alexandra Khartabil, David Howard

#### Department of Kinesiology, California State University San Bernardino, San Bernardino, CA, USA

##### **Correspondence:** Guillermo Escalante (gescalan@csusb.edu)

**Background**

Physique competitors must achieve low levels of body fat while maintaining as much lean body mass as possible to be successful in their sport. Research is lacking as to the principles and methods of resistance and cardiovascular training used by physique competitors as they prepare for a competition. This study investigated the resistance and cardiovascular training practices of in-season male physique competitors via a retrospective survey given one day prior to their competition. Furthermore, body composition, height, and weight were assessed.

**Materials and Methods**

Ten amateur male physique competitors (age = 30.0 +/- 5.8 yrs, height = 1.72 +/- .08 m, weight = 76.4 +/- 7.6 kg) volunteered for this study. A comprehensive survey that asked specific questions about training over the last 30 days was filled out in person via an online survey sent to their phones on Qualtrics.com before height, weight, and body composition were assessed. Height and weight were measured with an electronic scale and a standard stadiometer. Body fat was estimated with an A-mode ultrasound device (US) (BodyMetrix, IntelaMetrix, Brentwood, CA, USA) per the manufacturer recommended guidelines. The Jackson-Pollock 3-site formula was used to estimate body composition based on the body density prediction equation from the subcutaneous fat measurements of the chest, abdomen, and thigh as determined by the US device.

**Results**

Body fat percentage measures for the US were 7.2 ± 2.4. Table 1 summarizes the resistance and cardiovascular training practices of physique competitors.

**Conclusions**

Physique competitors follow various resistance training and cardiovascular training methods in order to achieve low levels of body fat while attempting to maintain their fat free mass. The results of our study suggest that more research is required to investigate the best methods necessary to improve body composition. Our study further suggests that current evidence based principles to improve body composition are not being implemented.

**Table 1 (abstract A56).** Resistance and Cardiovascular Training Practices of Physique Competitors

**Cardiovascular Training**		**Resistance Training**	
**Question**	**Results**	**Question**	**Results**
Fasted Cardio	60% yes 20% no 20% sometimes	Days per week resistance training	5/wk = 10% 6/wk = 50%, 7/wk = 20% 8+/wk = 20%
Days of Cardio per week with 10 minutes or more	1/wk = 10% 3/wk = 10% 4/wk = 10% 5/wk = 10% 6/wk = 10% 7/wk = 10% 10/wk = 20% 15/wk = 20%	Average minutes per resistance training session	60 min= 20% 75 min= 30% 90 min= 10% 120+ min=40%
Average minutes per cardio session	20 min= 20% 30 min= 10% 40 min = 30% 45 min= 30% 60 min = 10%	Upper body average rest between sets	30-60 sec = 60% 61-90 sec = 20% 91-119 sec = 20%
Weights and cardio together	Yes 70% No 10% Sometimes 20%	Lower body average rest between sets	30-60 sec = 50% 61-90 sec = 30% 91-119 sec = 20%
Low intensity cardio days per week (1-3 RPE)	1/wk =20% 2/wk = 30% 4/wk = 10% 5/wk = 20% 7/wk = 10% 9/wk = 10%	Training techniques used	Super Sets = 40% Drop sets = 20% Tri/giant sets = 10% Variable resistance = 10%, Eccentrics = 10%, Don’t know = 10%
Low-Mod cardio days per week (4-5 RPE)	1/wk = 50% 2/wk =20% 4/wk = 10% 7/wk = 20%	Use of Periodization	Blocked periodization= 20% Don’t know= 80%
Mod-High Cardio days per week (6-8 RPE)	1/wk = 10% 2/wk = 50% 5/wk = 10% 6/wk = 10%, 7/wk = 10% 15+/wk = 10%	% of sets taken to failure	Less than 10% =20% 11-19% = 20% 20-29% = 20% 60-69% = 10% 70-79 %= 10% 80-89 %= 10% 100 %= 10%
High cardio days per week (9-10 RPE)	1/wk = 60% 2/wk = 10% 5/wk = 20% 15+/wk = 10%		

## A57 Effects of creatine monohydrate plus beef protein on body composition and strength levels after 8-weeks of a cluster-based resistance training; a pilot study

### Diego A. Bonilla^1,2^, Salvador Vargas^3,4^, Jorge L. Petro^2,5^, Ramón Romance^4^, Manuel García^3^, Brad J. Schoenfeld^6^, Richard B. Kreider^7^ FISSN, Javier Benítez-Porres^4^

#### ^1^ Department of Genetics, Faculty of Science and Technology, University of the Basque Country (UPV/EHU), Leioa 48940, Spain; ^2^ Research Division, DBSS, Bogotá 110861, Colombia; ^3^ EADE-University of Wales Trinity Saint David, Málaga 29017, Spain ; ^4^ Human Kinetics and Body Composition Laboratory, University of Málaga, Málaga 29010, Spain; ^5^ Research Group in Physical Activity, Sports and Health Sciences, Universidad de Córdoba, Montería 230002, Colombia; ^6^ Department of Health Sciences, CUNY Lehman College, NY 10468, USA; ^7^ Exercise & Sport Nutrition Lab, Human Clinical Research Facility, Texas A&M University, College Station, TX 77843, USA

##### **Correspondence:** Diego A. Bonilla (dabonilla@g-se.com)

**Background**

Creatine monohydrate (CrM) supplementation has been reported to improve body composition and muscle strength when combined with resistance training. Cluster-based resistance training (CT), which involves the insertion of intraset pauses (IP), has been proposed as a method to enhance training adaptations. The purpose of this study was to determine if CrM supplementation in combination with consumption of a high-protein diet would augment the impact of CT thereby promoting greater improvements in lower-limb (LL) fat-free mass (FFM) and muscular strength.

**Materials and Methods**

Twenty four resistance-trained volunteeers (25.9±8.1 yr, 176.4±7.0 cm, 75.6±8.9 kg body mass) participated in this study. Subjects were allocated to one of three groups: CT and CrM (0.1 g/kg/d) + beef protein (0.5 g/kg/d) post-training supplementation (*n*=8, CT-CrM); CT only (*n*=8); or a control that followed their habitual diet and training program (*n*=7). Training included 8-weeks of LL-CT (twice per week with 72 hours recovery) consisting of 3 sets of squat, deadlift and leg press with 4 clusters of 3RM; 20 s of IP and 180 s interset. Subjects in CT-CrM and CT groups were instructed to consumed 2.5 g/kg/d of protein. LL-FFM (DXA) and muscle strength (back squat 1RM and CMJ) were measured at baseline and post-study. Mean changes from baseline were analyzed by one-way ANOVA. Dependent t-tests were used for pairwise comparisons.

**Results**

Statistical analysis (x̅ ± SD [CIs 95%], p value, Cohen’s *d* effect size) showed significant changes in LL-FFM in the CT-CrM (1.4±0.7 kg [0.8–2.1], p<0.01, 0.6) and CT group (0.9±0.5 kg [0.5–1.3], p=0.001, 0.2) from baseline, whereas non-significant changes were observed in control (0.5±1.1 kg [-0.6–1.5], p=0.001, 0.2). Back squat 1RM improved in CT-CrM (24.0±9.5 kg [15.2–32.8], p= 0.014, 1.5) and CT (14.5±12.3 kg [4.2–24.8], p=0.012, 0.8) but not in control (7.3±9.8 kg [-2.9–17.6], p=0.124, 0.5). CMJ improved in CT-CrM (1.8±1.5 kg [0.4–3.2], p=0.002, 1.0) and CT (0.9±0.5 kg [0.5–1.3], p=0.031, 0.5) but not in control (0.1±3.1 kg [-2.9–3.0], p=0.948, 0.0). Post hoc analysis revealed that LL-FFM tended to be greater between CT-CrM and control (p=0.074), while squat was significantly different (p=0.037).

**Conclusions**

This pilot study showed that CT-CrM and CT produced similar increases in LL-FFM and CMJ. However, there was some evidence that CT-CrM promoted greater adaptations in LL-FFM and squat 1RM strength compared to control. Future research should examine whether CrM supplementation augments CT.

**Acknowledgements** – Diego A. Bonilla serves as a Science Product Manager for MTX Corporation® (Europe). Brad J. Schoenfeld serves on the scientific advisory board for Dymatize Nutrition Corporation. This study was supported by University of Málaga (Campus of International Excellence Andalucía Tech) and MTX Corporation (Spain, Europe).

## A58 Effects of three Cluster-based resistance training protocols on strength and body composition in trained men maintaining a high protein diet

### Salvador Vargas^1,2^, Ramón Romance^1^, Brad J. Schoenfeld^3^, Manuel García^2^, Jorge L. Petro^4^, Diego A. Bonilla^5^, Richard B. Kreider, FISSN^6^, Fernando Martín^7^, Javier Benítez-Porres^1^

#### ^1^ Human Kinetics and Body Composition Laboratory, University of Málaga, Málaga 29010, Spain; ^2^ EADE-University of Wales Trinity Saint David, Málaga 29017, Spain; ^3^ Department of Health Sciences, CUNY Lehman College, NY 10468, USA; ^4^ Research Group in Physical Activity, Sports and Health Sciences, Universidad de Córdoba, Montería 230002, Colombia; ^5^ Department of Genetics, Faculty of Science and Technology, University of the Basque Country (UPV/EHU), Leioa 48940, Spain; ^6^ Exercise & Sport Nutrition Lab, Human Clinical Research Facility, Texas A&M University, College Station 77843, USA; ^7^ Research Unit in Sports and Health, University of Valencia, Valencia, Spain

##### **Correspondence:** Salvador Vargas (salvadorvargasmolina@gmail.com)

**Background**

The aim of this study was to investigate the effects of three cluster training (CT) protocols comprised of different intra-sets pause (PIntra) and blocks of repetitions (BK) on strength, power and body composition in individuals maintaining a high protein diet (~2.5 g·kg^-1^·d^-1^).

**Materials and Methods**

Twenty-nine resistance-trained men (26.9 ± 8 years; 176.2±8.4 cm; 75.5±9.7 kg; 24.2±2.0 kg∙m^-2^) were randomized to either a PIntra 20 s and BK 3 RM (n = 8, CL1), PIntra 40 s and BK 3 RM (n = 7, CL2), PIntra 20 s and BK 6 RM (n = 7, CL3) or a control group (n = 7, CG). All participants performed two sessions per week of resistance training (RT) for the lower limbs with 72 hours of recovery between sessions. Training sessions were supervised for all groups except the control. Participants were provided a 2-week familiarization period, and then performed the prescribed study protocol for 8-weeks. Control group followed their habitual nutrition and training program. Fat-free mass (FFM) in the lower limbs (LL), muscle power and strength were assessed by DXA, CMJ and back squat 1RM (SQ) using a linear encoder, respectively. Data were analyzed by a GLM with repeated measures. Post-hoc comparisons were performed with Bonferroni correction and effect size analyzed by partial eta squared.

**Results**

GLM analysis revealed significant time effects. Mean change analysis revealed no statistical differences in LL-FFM; however, the greatest change (x̅±SD, ICs 95%; p value) was observed in CL1 (0.9±0.5 kg, 0.5-1.3; p=0.001). Changes in CL2 (0.6±0.5 kg, 0.2-1.1; p=0.01) and CL3 (0.6±0.4 kg, 0.2-1.0; p=0.01) were also significantly greater than baseline. The change in LL-FFM for the CG group was not significant (0.4±1.1 kg, 0.6-1.5; p=0.32). In regard to strength, the CL1 group obtained the greatest change from baseline (14.5±12.3 kg, 4.2-24.8; p=0.01) in the SQ, with a large effect size (0.8). The changes in the SQ for CL2 and CL3 were significant (10.1±4.3 kg, 6.1-14.0; p=0.001 and 9.5±4.9 kg, 5.0-14.0; 0.002, respectively), with a medium effect size (0.6 and 0.5, respectively); however, in CON there was no significant change (9.0±9.0 kg, -0.4-18.5; p=0.06). In regard to muscular power, no statistical differences from baseline were found for the CMJ in any of the groups, nor did we observe any between-group interactions.

**Conclusions**

Resistance-trained men maintaining a high protein diet can achieve significant muscular adaptations when using a PIntra of ~20 s in CT protocols with 3 RM blocks in multiple joint exercises of the LL. No additional benefits are seen using longer rest intervals. From a practical standpoint, this finding indicates that a PIntra of ~20 s allows for greater efficiency.

**Acknowledgements**: This study was supported by University of Málaga (Campus of International Excellence Andalucía Tech).

## A59 An examination of a novel weight loss formula on measurements of body composition

### R. Sowinski, T. Grubic, R. Dalton, J. Schlaffer, AG. Reyes, V. Jenkins, S. Williamson, C. Rasmussen, P. Murano, M. Greenwood, FISSN, C.P. Earnest, FISSN, and R.B. Kreider, FISSN

#### Exercise & Sport Nutrition Lab, Texas A&M University, College Station, TX, USA

##### **Correspondence:** R.B. Kreider (rbkreider@tamu.edu)

**Background**

Studies from Africa have reported that *Dichrostachys glomerata* (DG) supplementation (200 – 400 mg/d) promotes significant reductions in weight and fat loss in obese individuals without exercise or diet intervention. This study investigated whether adding DG to weight loss supplements with and without caffeine promotes weight and fat loss in overweight individuals without exercise or dietary modification.

**Materials and Methods**

In a double-blind, parallel, stratified randomized, placebo-controlled trial; 68 men (n=31) and women (n=37), (37±5 yr; 88.9±16.6 kg; BMI 25.0-34.9 kg/m^2^; Fat 35.2±7.7%; Activity 6,857±1,512 steps/wk) supplemented their diet for 12 weeks with a placebo (PLA, 6 g dextrose), a weight loss formulation containing DG (300mg/d), Sensoril® (250mg/d; Ashwaganda/Withania somnifera), Bioperine (5mg/d), Capsimax® (50mg/d; 4% Capsaicinoids), Rhodiola rosea extract (60mg/d), L-Theanine (100mg/d), Clubmoss extract (CE) (5mg/d; 1% Huperzine), and Bacopa monneri extract (50mg/d) [WL] or DG (300mg/d), CE (10mg/d), XR Caffeine (150mg/d; 77% Caffeine), Caffeine-anhydrous (250mg/d; 98.5%), Sensoril® (125mg/d), Capsimax® (50mg/d) [WL+C]. DEXA body composition measurements were obtained at baseline and after 4, 8, and 12-weeks of supplementation. Data were analyzed using univariate and multivariate General Linear Model analysis with repeated measures and mean changes from baseline with 95% confidence intervals (CI).

**Results**

Overall Wilks' Lambda for Time (p=0.07) and Sex (p<0.01) effects for body composition were observed. Analysis of changes from baseline with 95% CI indicated a significant decrease from baseline in fat mass (WL -0.56±0.95 [-1.02, -0.14], WL -0.63±1.47 [-1.23, -0.02] kg) at wk4 and wk8, respectively. Body fat also decreased (WL -0.63±1.26 [-1.16, -0.10], WL -0.78±1.31 [-1.45, 0.07]%) at wk8 and wk12, respectively.

**Conclusions:**

Supplementation of a DG containing weight loss formulation was shown, with GLM, to have no significant differences between groups regarding body composition measures, using the current dose (300mg/1x/d). The addition of caffeine did not alter the outcome. Supplement groups showed initial minor weight loss as well as decreased FM and BF%, with indications of having greater effect on males. Further research is required to determine effective dose and paired with a diet and/or exercise program for functional assessment of weight loss potential.

**Acknowledgments**

This study was supported by Nutrabolt (Bryan, TX) through an unrestricted grant to Texas A&M University.

## A60 An examination of a novel weight loss formula on measurements of resting energy expenditure

### V. Jenkins, R. Sowinski, T. Grubic, R. Dalton, J. Schlaffer, AG. Reyes, S. Williamson, C. Rasmussen, P. Murano, M. Greenwood, CP. Earnest, and RB. Kreider

#### Exercise & Sport Nutrition Lab, Texas A&M University, College Station, TX, USA

##### **Correspondence:** RB. Kreider (rbkreider@tamu.edu)

**Background**

Studies from Africa have reported that *Dichrostachys glomerata* (DG) supplementation (200 – 400 mg/d) promotes significant reductions in weight and fat loss in obese individuals without exercise or diet intervention. This study investigated whether adding DG to weight loss supplements with and without caffeine affects resting energy expenditure (REE) in overweight individuals without exercise or dietary modification.

**Materials and Methods**

In a double-blind, parallel, stratified randomized, placebo-controlled trial; 68 men (n=31) and women (n=37), (37±5 yr; 88.9±16.6 kg; BMI 25.0-34.9 kg/m^2^; Fat 35.2±7.7%; Activity 6,857±1,512 steps/wk) supplemented their diet for 12 weeks with a placebo (PLA, 6 g dextrose), a weight loss formulation containing DG (300mg/d), Sensoril® (250mg/d; Ashwaganda/Withania somnifera), Bioperine (5mg/d), Capsimax® (50mg/d; 4% Capsaicinoids), Rhodiola rosea extract (60mg/d), L-Theanine (100mg/d), Clubmoss extract (CE) (5mg/d; 1% Huperzine), and Bacopa monneri extract (50mg/d) [WL] or DG (300mg/d), CE (10mg/d), XR Caffeine (150mg/d; 77% Caffeine), Caffeine-anhydrous (250mg/d; 98.5%), Sensoril® (125mg/d), Capsimax® (50mg/d) [WL+C]. REE measurements were obtained at baseline and after 4, 8, and 12-weeks of supplementation. Data were analyzed using univariate and multivariate General Linear Model analysis with repeated measures and mean changes from baseline with 95% confidence intervals (CI) after wk4, wk8, and wk12, respectively.

**Results**

Overall effects for Time (p<0.01), Group x Time (p=0.04), and Sex (p<0.01) effects for REE values were observed. REE significantly increased from baseline (PLA 162±277 [68, 266], PLA 135±310 [31, 248], WL+C 111±220 [10, 207] kcal/d) at wk4, wk8, and wk12, respectively. REE/kg also increased (PLA 1.84±2.65 [0.84, 2.88], PLA 1.38±2.75 [0.34, 2.49], WL+C 1.57±2.37 [0.5, 2.6] kcal/kg/d) at wk4, wk8, and wk12, respectively.

**Conclusions**

Supplementation of a DG containing weight loss formulation was shown, with GLM, to have no significant differences between groups regarding REE measures, using the current dose (300mg/1x/d). The addition of caffeine did not alter the outcome. Supplement groups showed increase in REE (kcal/d and kcal/kg/d) focused around wk8 and wk12, seemingly more so in females. However, the changes were not different from the changes seen in PLA. Further research is required to determine effective dose and paired with a diet and/or exercise program for functional assessment of metabolic aid potential.

**Acknowledgments**

This study was supported by Nutrabolt (Bryan, TX) through an unrestricted grant to Texas A&M University.

## A61 Cannabinoids and sports nutrition – is product marketing ahead of the science?

### Susan Hewlings, Douglas Kalman

#### Nutrasource. Guelph, ON, Canada

##### **Correspondence:** Douglas Kalman (dkalman@nutrasource.ca)

**Background**

Associated with the legalization of cannabis for medical and recreational purposes in various states across the United States, is a push for hemp and cannabis constituents, such as cannabidiol (CBD), to be part of the food, beverage and dietary supplement product supply. Although in review, currently it is not legal at the Federal level to include cannabis or any of its derivatives into food, beverages or dietary supplements. However, the enactment of the 2018 U.S. Farm Bill federally legalized hemp-derived CBD as long as it does not contain more than 0.3% tetrahydrocannabinol (THC). However, this does not mean that CBD can be added to the above-mentioned products. Further confusing the public is that while CBD is legal federally, not all states follow the federal law. CBD products are marketed for a variety of uses from relieving pain to better sleep, to enhanced cognition. Many products target the Sport and Exercise Nutrition market. The purpose of this brief review was to examine the advertising, regulatory and published science, that may substantiate claims made regarding the use of CBD in sports nutrition products.

**Materials and Methods**

Database searches using key terms.

**Results**

Using Google and the search term “CBD sports nutrition”, 15,400,000 responses populated, ranging from articles to products. Using the search term “cannabinoids sport nutrition” revealed 778,000 responses, also ranging from articles to products. Using the search term “cannabinoid sport nutrition” in Pubmed.gov revealed 2 articles on the topic, none of which applied an experimental study design. Interestingly, 1 analytical study did find that the dietary supplement methoxyisoflavone and its metabolites can induce false-positive results in urinary immunoassay screening tests for cannabinoids, which may be important for drug-tested athletes. A search of clinicaltrials.gov revealed 228 studies related to CBD or cannabinoids related to pharmacokinetics or disease potential related uses. Only 6 of the 228 studies were utilizing healthy subjects. As of May 25, 2019, there were 0 registered studies with CBD listed for use or evaluation in sports or exercise nutrition.

**Conclusions**

The evidence is overwhelming that products are being marketed without being regulatory compliant, and more centric to sports nutrition, that there are tens of CBD centric marketed sports nutrition products, however there appears to be zero actual scientific direct evidence to support the products and claims in the intended population at this time. It is clear that safety and efficacy studies of CBD applicable to sport nutrition are needed.

## A62 Post-competition physiological adaptations in physique athletes: A case series

### Jaymes Longstrom, Lauren Colenso-Semple, Eric T. Trexler, Brian Waddell, Sarah Ford, Kait Callahan, Tu Nguyen, Bill I. Campbell

#### Performance & Physique Enhancement Laboratory, University of South Florida, Tampa, FL, USA

##### **Correspondence:** Bill I. Campbell (bcampbell@usf.edu)

**Background**

The purpose of this study was to investigate physiological changes that occur in natural physique sport athletes during the post-competition period.

**Materials and Methods**

Participants included three male (34.3±6.8 years, 181.6±8.9 cm) and four female (29.3±4.9 years, 161.4±6.0 cm) natural physique athletes. Body composition (fat mass [FM]and fat-free mass [FFM]; Skinfold) and resting metabolic rate (RMR; indirect calorimetry) were assessed 1-week prior to competition, followed by 4 weeks and 8-10 weeks post-competition. Blood hormones (free triiodothyronine (T3), free thyroxine (T4), and leptin) were assessed at 1-week prior to competition and 8-10 weeks post-competition. All assessments were performed following an 8-hour overnight fast. Participants tracked macronutrient intake daily for the duration of the study. Data were analyzed via one-tailed Wilcoxon-Pratt Signed-Rank tests and Kendall’s Rank Correlation tests at the α=0.05 significance level.

**Results**

At the group level, significant (p<0.05) increases were observed for bodyweight, FM, bodyfat%, RMR, and blood hormones (T3, T4, and leptin). Fat-free mass increased to a non-significant degree, but a trend was observed (p = 0.055). Observed changes varied substantially between subjects; raw values of key variables are presented for each participant in Table 1. Percent change (%△) in body weight was associated with △RMR (tau (τ) =0.62; p=0.03) and △leptin (τ =0.59; p=0.03). △FM% was associated with △RMR (τ =0.90; p=0.001) and △leptin (τ =0.68; p=0.02). △bodyfat % was associated with △leptin (τ =0.88; p=0.003). △leptin was associated with △RMR (τ =0.59; p=0.03).

**Conclusions**

To the best of our knowledge, this is the first case series investigating physiological outcomes associated with weight gain in natural physique athletes during the post-competition period. The primary observations from this study included group-level increases in adiposity, blood hormones, and RMR; substantial between-participant variability; and strong associations between changes in adiposity, leptin, and RMR.

**Table 1 (abstract A62).** Raw values for bodyfat % (skinfold), RMR (kcal/day), leptin (ng/mL), T3 (pg/mL), and T4 (ng/dL) at pre-competition (1-week prior to contest) and 8-10-weeks post-competition.

	**Bodyfat %**		**RMR**		**Leptin**		**Free, T3**		**Free, T4**	
Participant	Pre	Post	Pre	Post	Pre	Post	Pre	Post	Pre	Post
Male 1	7.6	10.8	1,852	2,102	0.3	1.0	2.7	2.7	1.1	1.1
Male 2	7.0	9.2	1,660	1,667	0.5	0.3	1.8	1.8	0.9	0.9
Male 3	6.3	8.2	1,601	1,765	0.8	0.7	1.9	2.4	0.8	1.0
Female 1	15.8	27.1	1,264	1,536	0.5	3.7	1.6	2.9	0.8	0.9
Female 2	12.5	15.3	1,471	1,381	0.4	1.0	1.5	2.4	0.8	1.1
Female 3	14.6	20.4	1,259	1,769	1.3	2.4	2.8	3.0	1.1	1.2
Female 4	16.6	22.0	1,393	1,447	1.0	1.7	2.5	3.0	1.0	1.1

**Acknowledgements:**

Hormone testing was supported by BioLayne, LLC and ProPhysique Inc.

## A63 Changes in resting cortisol following eight weeks of high- or moderate-volume resistance training

### Lauren M. Colenso-Semple, Samuel L. Buckner, Jaymes M. Longstrom, Megan Humphries, Wenyuan G. Zhu, Brian Waddell, Noam Yitzchaki, Traci Smith, Eric T. Trexler, Bill I. Campbell

#### Performance & Physique Enhancement Laboratory, University of South Florida, Tampa, FL, USA

##### **Correspondence:** Bill I. Campbell (bcampbell@usf.edu)

**Background**

Cortisol is the major glucocorticosteroid hormone produced in the adrenal cortex, and is a key regulator of metabolism, immune function, and physiological response to stress. Non-functional overreaching is characterized by inadequate recovery from prolonged high-volumes of exercise, which ultimately impairs performance. The purpose of the study was to compare changes in resting cortisol levels in young healthy females in response to 8 weeks of high-volume or moderate-volume resistance training.

**Materials and Methods**

Thirty-six resistance-trained women (mean ± SD: Age: 23 ± 4 yrs; Height: 63.7 ± 2.5 in; Weight: 134 ± 20 lbs) were assigned to a high-volume (HV) (n=17) or moderate-volume (MV) (n=19) experimental group. Participants trained on 3 non-consecutive days per week for 8 weeks with a total of 81 weekly sets (HV) or 36 weekly sets (MV) of lower-body exercises. Each session consisted of 5 sets (HV) or 2 sets (MV) of barbell squats, stiff-leg deadlifts, and barbell hip thrusts, and 4 sets (HV) or 2 sets (MV) of knee extension, leg curl, and cable abduction. The target repetition ranges were specified as 6-8 (Day 1), 8-10 (Day 2), and 12-15 (Day 3). Loads were adjusted to ensure each set was terminated 2 repetitions from failure. All sessions were supervised by certified personal trainers. Resting salivary cortisol was assessed at baseline and 48-72 hours following the final training session. Samples were tested for salivary cortisol using a high sensitivity enzyme immunoassay (Cat. No. 1-3002). Data were analyzed using ANCOVA on change scores with group as the predictor variable and pre-test value as a covariate to adjust for baseline values. ANCOVA results are presented as least square mean ± standard error.

**Results**

Salivary cortisol decreased after 8 weeks of training (HV = -1.3 nmol/L ± 1.45, MV = -3.2 nmol/L ± 1.37) with no statistically significant differences between groups (p = 0.36). The overall change for the full sample (collapsed across groups) was -2.3 nmol/L (-14%), with a pre-test range of 5.6 - 34.7 nmol/L and a post-test range of 4.2 - 33.2 nmol/L.

**Conclusions**

The results indicate a similar decrease in resting cortisol following 8 weeks of high-volume and moderate-volume resistance training. While the analysis did not reveal significant differences at the group level, there were notable interindividual differences in change in cortisol level from pre to post-intervention, ranging from -23.8 to +15.2 nmol/L.

**Acknowledgement**

This study was funded by a grant from the College of Education at the University of South Florida.

## A64 Sex differences in rate of recovery in single-joint resistance exercise performance

### Megan Humphries^1^, Madelin Siedler, Priscila Lamadrid, Sarah Ford, Traci Smith, Gillian SanFilippo, Maria De La Torre, Brian Waddell, Benjamin Miller, Noam Yitzchaki, Lauren Colenso-Semple, Eric Trexler, Samuel Buckner, Bill I. Campbell

#### Performance & Physique Enhancement Laboratory, University of South Florida, Tampa, FL, USA

##### **Correspondence:** Bill I. Campbell (bcampbell@usf.edu)

**Background**

Some research reports sex-differences in rate of performance recovery from resistance training, indicating that females recover faster than males, and can therefore train more frequently. However, this observation may be unique to the exercise/modality tested. The purpose of this study was to evaluate sex differences in the rate of recovery in the performance of upper- and lower-body single-joint dynamic exercises.

**Materials and Methods**

29 resistance-trained males (n=17) and females (n=12) completed a repeated measures, randomized, parallel groups design comparing sex differences in rate of recovery. The protocol consisted of a baseline performance assessment, a recovery period (4 hours, 24 hours, or 48 hours—order was randomly assigned) and a post-recovery performance assessment. The baseline and post-recovery assessments were identical and consisted of four sets of 10 repetition maximum (RM) bicep curls and four sets of 10RM knee extensions to failure with 90 seconds of rest between each set. “Recovery” was defined as the number of total repetitions completed in the post-recovery bout, expressed as a percentage of baseline repetitions. Data were analyzed using a series of general linear models at a significance level of α=0.05, and presented as mean ± standard deviation.

**Results**

Bicep Curl: Time × sex interaction (p=0.75), main effect for time (p=0.11), and main effect for sex (p=0.59) did not reach statistical significance. Specifically, performance recovery was 99.8 ± 7.7% at 4 hrs, 94.2 ± 8.0% at 24 hrs, and 100 ± 8.1% at 48 hrs. Leg Extension: Time × sex interaction (p=0.30) and main effect for sex (p=0.86) were not statistically significant. A trend was observed for the main effect of time (p=0.06), with the lowest values observed at the 4-hour time point (98.1 ± 13.4% at 4 hrs, 105.6 ± 13.5% at 24 hrs, 101.7 ± 10.4% at 48 hrs). Exploratory models including key demographic and body composition variables as covariates yielded similar results. *Table 1* provides an overview of the data for each recovery period for males and females.

**Conclusions**

Performance recovery rates for both exercises were similar in males and females, indicating that sex does not dictate the rate of recovery from single-joint dynamic resistance exercises.

These findings may not be applicable to multi-joint exercises and/or those requiring greater technical skill. Future research should investigate the rate of performance recovery from a variety of exercises in a single sample.

**Table 1 (abstract A64).** Performance recovery (total repetitions completed) in males and females for biceps curl and leg extension.

Group	Baseline (4-Hour)	4-Hour Recovery	Baseline (24-Hour)	24-Hour Recovery	Baseline (48-Hour)	48-Hour Recovery
Biceps Curl (reps)						
Males	30.3 ± 8.4	30.0 ± 7.2	31.5 ± 8.8	29.8 ± 8.6	30.8 ± 9.0	30.8 ± 8.8
Females	35.1 ± 7.9	35.3 ± 7.3	35.6 ± 7.1	33.4 ± 7.5	34.1 ± 6.5	34.3 ± 8.0
Leg Extension (reps)						
Males	38.7 ± 9.3	37.6 ± 7.9	37.6 ± 7.4	38.5 ± 6.4	37.4 ± 7.8	38.9 ± 8.1
Females	42 ± 9.9	40.4 ± 8.8	37.8 ± 6.2	40.8 ± 7.7	41.2 ± 8.7	40.2 ± 8.2

## A65 Effects of caffeine vs. caffeine plus TeaCrine® administration on multiple indices of cognitive function in healthy men and women

### Kyle R Cesareo^1^, Betsy J Raub^1^, Jennifer E Sandrock^1^, Chad M Kerksick^2^, Hector L Lopez^1^, Tim N Ziegenfuss^1^

#### ^1^The Center for Applied Health Sciences, Canfield, OH 44406, USA; ^2^Exercise and Performance Nutrition Laboratory, School of Health Sciences, Lindenwood University, St. Charles, MO, USA

##### **Correspondence:** Tim N Ziegenfuss (TZ@appliedhealthsciences.org)

**Background**

Theacrine (1,3,7,9-tetramethyluric acid) is a naturally occurring purine alkaloid present in certain teas, coffees*,* and botanical sources that regulates brain glucose metabolism, inhibits phosphodiesterases, and exhibits anti-adenosinergic, dopaminergic, anti-inflammatory, and analgesic properties. Previous research has documented increased feelings of energy, reduced fatigue, and powerful influences on improving focus, concentration, and motivation. Limited research has examined the potential additive effects of theacrine + caffeine on sustained cognitive performance. The purpose of this study was to compare cognitive performance during caffeine vs. caffeine + TeaCrine® ingestion using validated, objective measurements of cognitive function and subjective, qualitative measures of various neuromotor/cognitive qualities.

**Materials and Methods**

Using a randomized, double-blind, within-subject (crossover) design, 8 men and 4 women (mean ± SD age, height, weight: 21.9 ± 2.6 yr, 174.2 ± 4.4 cm, 76.5 ± 7.0 kg) completed three counterbalanced trials: 300 mg Caffeine (CAF), placebo (PLA), and 300 mg Caffeine + 62.5 mg Teacrine**®** (COMBO). Before (baseline), 60 and 180 minutes after ingesting their respective supplement, all subjects completed 5 consecutive STROOP tests (5x45-sec interspersed with 60 sec rest) to assess executive function, cognitive flexibility, mental acuity, selective attention, and processing speed as well as Visual Analogue Scales (VAS) for perceived energy, mood, focus, concentration, and motivation at each hour (i.e. baseline, 60, 120, 180 and 240 min post-ingestion). Data were analyzed via ANOVA, t-tests (p<0.05) and effect sizes (ES).

**Results**

During COMBO, 11/12 (92%) of subjects scored >60 for total STROOP score vs. 2/12 (17%) during CAF (p<0.01). Significantly greater number of correct STROOP responses were noted during COMBO (+4.4 ± 3.5) in comparison to CAF (+0.83 ± 4.1, p=0.031) at 180 min. Additionally, the time per score during the STROOP test was significantly improved only during COMBO at 60 min (0.78 ± 0.11 sec) and 180 min (0.77 ± 0.11 sec, both p=0.005). At 240 min post-ingestion, VAS for energy (+6.2 ± 1.9, p=0.027) and concentration (+6.4 ± 1.5, p=0.044) improved in COMBO only, and motivation was greater in COMBO (+1.8 ± 1.3) vs. CAF (+0.17 ± 2.0, p=0.045).

**Conclusions**

Collectively, these findings indicate that adding 62.5 mg of TeaCrine® to 300 mg of caffeine leads to enhanced benefits in attention, executive/cognitive function and processing speed as well as subjective improvements in energy, motivation and concentration. These benefits appeared within 60 minutes and continued to improve in magnitude over the 3-4 hour data acquisition period.

**Acknowledgements**

This study was funded in part by a research grant from Compound Solutions, Inc (Carlsbad, CA). HLL and TNZ are co-inventors for the intellectual property covering the uses of theacrine (TeaCrine®). The researchers in this study independently collected, analyzed, and interpreted the results without input from Compound Solutions, Inc. All other authors declare no conflict of interest.

## A66 Effects of a CBD-containing supercritical fluid extract of hemp on markers of optimal wellness, stress resilience, and recovery in healthy subjects

### Hector L Lopez^1^, Tim N Ziegenfuss^1^, Kyle R Cesareo^1^, Betsy J Raub^1^, Jennifer E Sandrock^1^, Chad M Kerksick^2^

#### ^1^The Center for Applied Health Sciences, Canfield, OH 44406, USA; ^2^Exercise and Performance Nutrition Laboratory, School of Health Sciences, Lindenwood University, St. Charles, MO, USA

##### **Correspondence:** Hector L Lopez (hlopezmd@gmail.com)

**Background**

The endocannabinoid system (ECS) is a master endogenous homeostatic system consisting of 1) lipid based signaling compounds (endocannabinoid ligands), 2) specialized cannabinoid receptors found throughout most tissues in the body and 3) biosynthetic and catabolic enzymes that regulate the endogenous ligands. Through both direct and indirect actions, endocannabinoids modulate and influence a variety of physiological systems, including pain, inflammation, thermoregulation, appetite, energy balance, muscle control/ coordination, sleep health, stress responses, motivation, mood, and memory. There is a wide variety of *Cannabis sativa* L. cultivars with a complex phytochemical profile containing terpenophenolic cannabinoids and 400+ constituents that are distinguished by their chemical and genetic profile. Hemp is generally characterized as a cultivar of *C. sativa* whose predominant cannabinoid is cannabidiol (CBD), with a relatively low level of delta-9 tetrahydrocannabinol (THC) when assayed on a dry weight basis. Herein, we report on the psychometric indices of sleep, appetite, quality of life, and biomarkers of safety from supplementation with a CBD containing supercritical CO_2_ extract of the aerial parts of hemp.

**Materials and Methods**

Using a randomized, placebo-controlled, double-blind design, 65 overweight, but otherwise healthy men (n = 32) and women (n = 33) (mean ± SD age, BMI: 35.2 ± 11.4 yr, 28.5 ± 3.3 kg/m^2^) ingested either Hemp Oil Extract [HEMP, 60 mg/d PlusCBD Oil^TM^ (15 mg hemp-derived CBD)] or a placebo (PLA) every day for six weeks before their evening meal. Subjects followed their normal diet and a routine of low intensity physical activity (30 min of walking exercise 5 days per week). Outcome variables included changes in stress resilience, a 14-item panel of various psychometric parameters, heart-rate variability (LF, HF, LF/HF ratio, rMSSD), plasma chromogranin A, body composition (lean mass, fat mass, bone mineral content, VAT fat via DEXA) as well as general markers of health (heart rate, blood pressure, and comprehensive clinical chemistry panels of serum and plasma) before and after six weeks of supplementation. Data were analyzed via ANOVA, t-tests (p<0.05) and effect sizes (ES).

**Results**

Preliminary analyses revealed significant decreases in appetite (-6.2%, p=0.04, ES=0.22) and improvements in sleep quality (+22.0%, p=0.009, ES=0.54), sleep quantity (+21.3%, p=0.02, ES=0.58) and pleasure from life (+12.5%, p=0.006, ES=0.46) in HEMP only. All values for hepato-renal function (AST, ALT, BUN, creatinine, total bilirubin, alkaline phosphatase), cardiovascular health (heart rate, blood pressure), fasting blood lipids (cholesterol, triglycerides, HDL, LDL) whole blood cell counts (hemoglobin, hematocrit, RBC, MCV, MCH, MCHC, RDW, differential white cell counts) remained within normal clinical limits, and no between-group differences over time were noted.

**Conclusions**

Collectively, these seminal findings in healthy subjects indicate that six weeks of HEMP PlusCBD Oil^TM^ supplementation can improve measures of sleep homeostasis, reduce appetite, and enhance quality of life. Ongoing and future analyses will examine changes in stress resilience, autonomic nervous system function, body composition, inflammatory cytokines, adipokines, as well as targeted gene expression/transcriptome (NFkB, NLRP3, UCP, PGC1a), etc.

**Acknowledgements**

This study was funded in part by a research grant from CV Sciences Inc (Las Vegas, NV). HLL and TNZ are members of the Advisory Board of CV Sciences. The researchers in this study independently collected, analyzed, and interpreted the results without input from CV Sciences, Inc. All other authors declare no conflict of interest.

